# Lattice Dynamics of Quasi-2D Perovskites from First Principles

**DOI:** 10.1021/acs.jpcc.4c01633

**Published:** 2024-07-16

**Authors:** Emily Y. Chen, Bartomeu Monserrat

**Affiliations:** † Cavendish Laboratory, University of Cambridge, J. J. Thomson Avenue, Cambridge CB3 0HE, United Kingdom; ‡ Department of Materials Science and Engineering, Stanford University, 496 Lomita Mall, Stanford, California 94305, United States; § Department of Materials Science and Metallurgy, University of Cambridge, 27 Charles Babbage Road, Cambridge CB3 0FS, United Kingdom

## Abstract

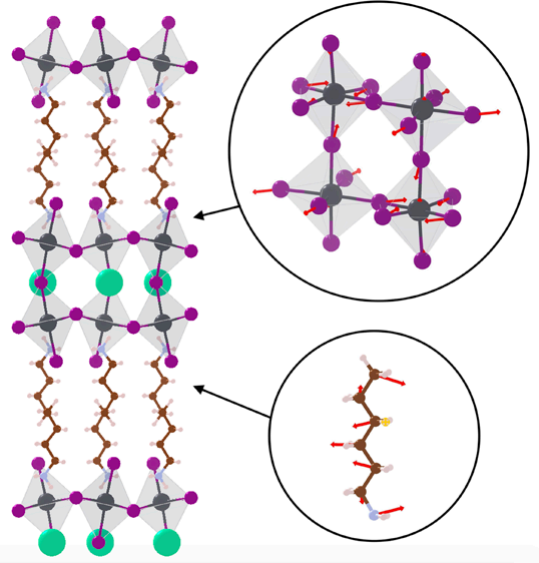

We present the vibrational properties and phonon dispersion for quasi-2D hybrid organic–inorganic perovskites (BA)_2_CsPb_2_I_7_, (HA)_2_CsPb_2_I_7_, (BA)_2_(MA)Pb_2_I_7_, and (HA)_2_(MA)Pb_2_I_7_ calculated from first principles. Given the highly complex nature of these compounds, we first perform careful benchmarking and convergence testing to identify suitable parameters to describe their structural features and vibrational properties. We find that the inclusion of van der Waals corrections on top of generalized gradient approximation (GGA) exchange-correlation functionals provides the best agreement for the equilibrium structure relative to experimental data. We also investigate the impact of the molecular orientation on the equilibrium structure of these layered perovskite systems. Our results suggest ground state ferroelectric alignment of molecular dipoles in the out-of-plane direction is unlikely and support the assignment of the centrosymmetric space group for the low-temperature phase of (HA)_2_(MA)Pb_2_I_7_. Finally, we compute vibrational properties under the harmonic approximation. We find that stringent energy cut-offs are required to obtain well-converged phonon properties, and once converged, the harmonic approximation can capture key physics for such a large, hybrid inorganic–organic system with vastly different atom types, masses, and interatomic interactions. We discuss the obtained phonon modes and dispersion behavior in the context of known properties for bulk 3D perovskites and ligand molecular crystals. While many vibrational properties are inherited from the parent systems, we also observe unique coupled vibrations that cannot be associated with vibrations of the pure constituent perovskite and ligand subphases. Energy dispersion of the low energy phonon branches primarily occurs in the in-plane direction and within the perovskite subphase and arises from bending and breathing modes of the equatorial Pb–I network within the perovskite octahedral plane. The analysis herein provides the foundation for future investigations on this class of materials, such as exciton–phonon coupling, phase transitions, and general temperature-dependent properties.

## Introduction

Layered (quasi-2D) halide perovskites, a subclass of hybrid organic–inorganic halide perovskites, are being extensively investigated for a diverse set of optoelectronic applications. Compared to 3D perovskites, which require anhydrous synthesis and operating conditions, layered halide perovskites have the advantage of being stable in ambient humidity,^1,2^ making them attractive options for light-emitting diodes and ferroelectric memory devices.^3−7^ They are also more compositionally flexible than their bulk counterparts, with the organic spacers, the inorganic framework, and the number of octahedral layers all representing possible pathways for compositional engineering.^3−5,8−10^ Finally, because the inorganic (perovskite) and organic (ligand bilayer) components form a natural quantum well system, layered halide perovskites exhibit unusually high exciton binding energies and represent a tunable platform to study exciton physics.^11,12^ Layered halide perovskites have also been explored in the context of spintronics and chiral photonics.^7,13^ However, because layered perovskites are structurally complex, how lattice vibrations modulate thermal and electronic properties of interest is much less understood in layered perovskites than in their 3D counterparts. While some experimental studies have probed electron–lattice or exciton-lattice interactions,^13−17^ theoretical work, especially *ab initio* work, has been relatively rare.

In this Article, we study the vibrational properties and phonon dispersion of layered perovskites from first-principles, building a foundation for future theoretical work on electron–lattice interactions. We consider two systems which have been studied experimentally, (BA)_2_(MA)Pb_2_I_7_ and (HA)_2_(MA)Pb_2_I_7_. Their structures resemble “slabs” of bulk perovskite spliced by bilayers of long organic ligands. Both systems consist of two layers of corner-sharing MX_6_ perovskite octahedra *per* organic ligand bilayer and are often referred to as *n* = 2 perovskites (where *n* is number of consecutive octahedral layers) in the literature. We focus on *n* = 2 perovskites because they are the smallest category of layered perovskites which retain the small cations at their prototypical bulk perovskite positions in between the two octahedral layers. As such, the inorganic subphase of *n* = 2 perovskites more closely resembles the bulk perovskite structure compared to *n* = 1 perovskites, which do not contain small cations at all. We henceforth refer to the small cations as occupying the “A” sites and the ligands as occupying the “R” sites.

Given the highly complex nature of these systems, we first perform careful benchmarking and convergence testing to identify suitable parameters to describe their structural features and vibrational properties. We find that the inclusion of van der Waals corrections on top of generalized gradient approximation (GGA) exchange-correlation functionals provides the best agreement for the equilibrium structure relative to experimental data. We also investigate the impact of the molecular orientation on the equilibrium structure of these layered perovskite systems. Our results suggest ground state ferroelectric alignment of molecular dipoles in the out-of-plane direction is unlikely and support assignment of the centrosymmetric space group for the low-temperature phase of (HA)_2_(MA)Pb_2_I_7_. Finally, we compute vibrational properties under the harmonic approximation. We find that stringent energy cut-offs are required to obtain well-converged phonon properties, and once converged, the harmonic approximation can capture key physics for such a large, hybrid inorganic–organic system with vastly different atom types, masses, and interatomic interactions. We discuss the obtained phonon modes and dispersion behavior in the context of known properties for bulk 3D perovskites and ligand molecular crystals. While many vibrational properties are inherited from the parent systems, we also observe unique coupled vibrations not consistent with vibrations of the pure constituent perovskite and ligand subphases. Dispersion of the low energy phonon branches primarily occurs in the in-plane direction and within the perovskite subphase, with minimal interlayer coupling. We expect the analysis herein provides a crucial foundation for future theoretical investigations on this class of materials, such as exciton–phonon coupling, phase transitions, and general temperature-dependent properties.

## Methodology

**Figure 1:**
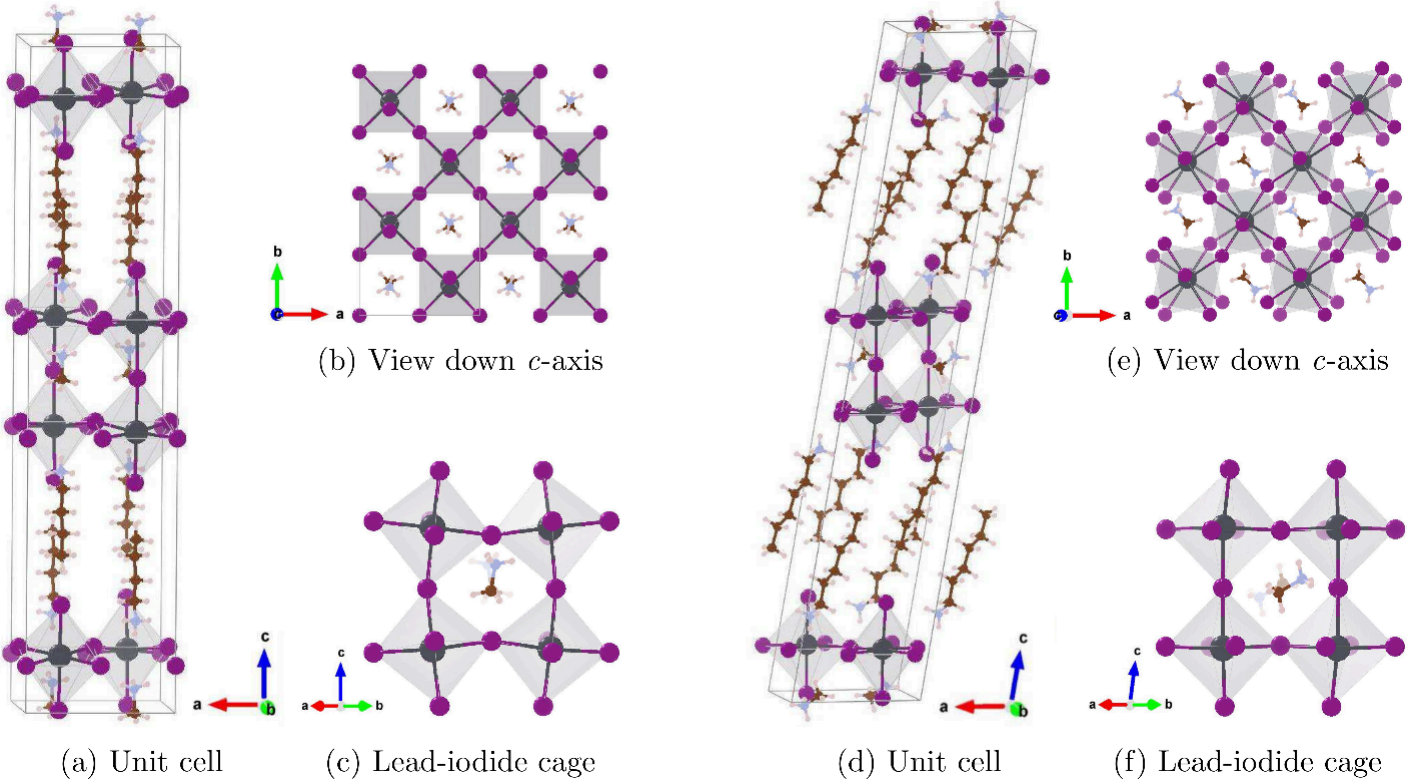
Crystal structure for (left) orthorhombic (BA)_2_(MA)Pb_2_I_7_ and (right) monoclinic (HA)_2_(MA)Pb_2_I_7_. Colors are purple (I), gray (Pb), brown (C), pink (N), and white (H).

Our starting points are crystallographic data from X-ray diffraction measurements. We obtain data for (BA)_2_(MA)Pb_2_I_7_ from ref (8) and for (HA)_2_(MA)Pb_2_I_7_ from ref (18). In both systems, methylammonium (MA) cations occupy the A sites within the perovskite layers. However, the former system has *n*-butylammonium (BA) cations separating the perovskite slabs, while the latter system has *n*-hexylammonium (HA) cations separating the perovskite slabs. Further, the BA-based perovskite crystallizes in an orthorhombic space group while the HA-based perovskite crystallizes in a monoclinic space group. This is because for layered perovskites containing primary alkylammonium cations, the phase transition temperature from monoclinic to orthorhombic increases with the length of the carbon chain. As such, even though the HA-based crystal is monoclinic at ambient temperature, it transitions to orthorhombic around 385 K.^5,19^ The unit cells are illustrated in Figure 1. Due to the dynamical rotational disorder of MA, we enumerate four configurations meant to represent the “extremes” of the possible MA dipole orientations, which are detailed in the Supporting Information. We also construct structural models where the MA molecules were replaced with Cs atoms, i.e., (BA)_2_CsPb_2_I_7_ and (HA)_2_CsPb_2_I_7_. This is a common procedure in theoretical calculations of hybrid organic–inorganic perovskites to reduce the computational complexity associated with the orientational disorder of MA cations.^20^

We perform electronic structure calculations within the Kohn–Sham density functional theory (DFT) framework^21,22^ in CASTEP^23^ using ultrasoft pseudopotentials.^24^ We use the Cs-based perovskite models to benchmark the performance of three different exchange-correlation functionals, namely, the Perdew–Zunger local density approximation (LDA),^22,25,26^ the Perdew–Burke–Ernzerhof (PBE) functional,^27^ and the modified Perdew–Burke–Ernzerhof functional for solids (PBEsol),^28^ with the latter two falling under the broader category of generalized gradient approximation functionals. We additionally test four semiempirical dispersion correction schemes, namely, those by (i) Ortmann, Bechstedt, and Schmidt^29^ (OBS), (ii) Grimme^30^ (G06), (iii) Tkatchenko and Scheffler^31^ (TS), and (iv) a many-body scheme by Tkatchenko and co-workers^32,33^ (MBD).

For all geometry optimization calculations, we use a kinetic energy cutoff of 600 eV for the plane wave basis set and a spacing of 2π × 0.04 Å^–1^ between **k**-points for the Γ-centered Monkhorst–Pack mesh for Brillouin zone sampling (corresponding to a 3 × 2 × 3 grid). For the Cs-based structural models, we constrain the symmetry (but not the cell dimensions) based on the known experimental crystal structures. For the MA-based structural models, since our choice of MA orientations necessitates breaking the known crystal symmetry, we do not apply any constraints. Convergence is reached when (i) all lattice parameters and angles are converged within 0.01 Å and 0.01 degrees, (ii) the maximum force on any ion is less than 0.01 eV/Å, and (iii) components of the stress tensor are smaller than 0.1 GPa.

We then choose (HA)_2_CsPb_2_I_7_ as our model system to study vibrational properties under the harmonic approximation. Lattice dynamics are simulated using the direct method^34^ with a displacement amplitude of 0.06 bohr. In calculating the harmonic force constants, we employ code from ref (35) and compute DFT forces from the PBEsol^28^ level with dispersion corrections by Tkatchenko and Scheffler.^31^ These DFT calculations are done with a plane wave cutoff of 800 eV and a Γ-centered Monkhorst–Pack mesh of spacing 2π × 0.04 Å^–1^. After converging the zone-center phonons, we construct supercells to explore phonon dispersion in the in-plane and out-of-plane directions. The former probes the two-dimensional nature of the phonons in the perovskite subphase, while the latter probes any interlayer coupling across stacked perovskite subphases. We also compute phonon density of states for both MA- and Cs-based systems, (HA)_2_(MA)Pb_2_I_7_ and (HA)_2_CsPb_2_I_7_, with explicit sampling of the dynamical matrix at the Γ-point only. Finally, we report IR spectra, computed using density-functional perturbation theory in CASTEP.^36^

## Results and Discussion

### Structural Properties

We present lattice parameters obtained with different exchange-correlation functionals and semiempirical dispersion correction schemes for (BA)_2_(Cs)Pb_2_I_7_ in Table 1 and (HA)_2_(Cs)Pb_2_I_7_ in Table 2. These are compared to experimental measurements for analogous BA/MA^8,19^ and HA/MA systems.^18,37^ A schematic illustrating the structural features used to compare the performance of the functionals can be found in the Supporting Information.

**Table 1:** Lattice Parameters and Select Structural Features for (BA)_2_(Cs)Pb_2_I_7_

compound			(BA)_2_(Cs)Pb_2_I_7_								(BA)_2_(MA)Pb_2_I_7_	
level of theory			LDA	PBE	PBEsol	LDA+OBS	PBE+G06	PBE+TS	PBEsol+TS	PBE+MBD	expt^a^	expt^b^
lattice parameters	*a* (Å)		8.022	8.715	8.468	7.428	8.415	8.468	8.230	8.588	8.8589(6)	8.8533(8)
	*b* (Å)		9.012	9.198	9.019	9.145	9.134	9.017	9.013	9.042	8.9470(4)	8.9317(8)
	*c* (Å)		40.087	43.184	41.563	39.657	41.350	40.933	40.701	41.610	39.347(2)	39.277(4)
	α (deg)		90	90	90	90	90	90	90	90	90	90
	β (deg)		90	90	90	90	90	90	90	90	90	90
	γ (deg)		90	90	90	90	90	90	90	90	90	90
	unit cell vol. (Å^3^)		2897.76	3461.7	3174.17	2694.03	3178.43	3125.63	3019.05	3231.29	3118.7	3105.9
bond angles (deg)	axial	avg	153.2	157.5	156.5	152.1	158.9	156.2	154.2	158.1	165.2(4)	d
		RMSE	11.9	7.6	8.6	13.1	6.2	9.0	11.0	7.0	d	d
	equatorial	avg	156.8	158.2	159.3	148.6	157.8	170	162.5	163.6	167.6(3)	d
		RMSE	11.1	9.8	8.6	19.1	10.4	7.3	6.3	5.0	d	d
bond lengths (Å)	axial (external)	avg	3.058	3.153	3.089	3.08	3.113	3.093	3.079	3.114	3.077(7)	d
		RMSE	0.019	0.076	0.012	0.007	0.036	0.016	0.002	0.037	d	d
	axial (internal)	avg	3.19	3.341	3.246	3.187	3.299	3.249	3.211	3.313	3.264(8)	d
		RMSE	0.076	0.078	0.024	0.078	0.038	0.022	0.055	0.052	d	d
	equatorial	avg.	3.087	3.222	3.143	3.052	3.160	3.126	3.100	3.154	3.168(13)	d
		RMSE	0.081	0.055	0.025	0.117	0.011	0.042	0.070	0.014	d	d
octahedral tilt (deg)	tilt in (110) plane		23.3	21.0	20.3	21.0	21.5	11.1	13.2	14.4	13.6^c^	d
	rotation about [001]		0.09	0.00	0.01	0.58	0.01	0.00	0.01	0.00	0.04	d
interplane distance (Å)	Pb–Pb across bilayer		13.83	15.08	14.44	13.71	14.23	14.01	14.02	14.29	13.20	d
	ligand N–perovksite I		0.89	0.72	0.85	0.58	0.89	1.17	0.96	0.99	0.81	d

a Measured at 298 K by ref (8).

b Measured at 300 K by ref (19).

c No standard deviation reported; data taken from crystallographic.cif file.

d Not reported.

**Table 2:** Lattice Parameters and Select Structural Features for (HA)_2_(Cs)Pb_2_I_7_

compound			(HA)_2_(Cs)Pb_2_I_7_								(HA)_2_(MA)Pb_2_I_7_	
level of theory			LDA	PBE	PBEsol	LDA+OBS	PBE+G06	PBE+TS	PBEsol+TS	PBE+MBD	expt^a^	expt^b^
lattice parameters	*a* (Å)		8.4728	8.8683	8.6355	8.6709	8.6117	8.4756	8.5098	8.6327	8.695(3)	8.8062(8)
	*b* (Å)		8.4839	8.9432	8.7090	7.9014	8.7522	8.8923	8.6053	8.8362	8.814(3)	8.9209(2)
	*c* (Å)		44.016	50.720	46.930	42.228	45.361	45.238	44.958	45.778	45.146(16)	45.3552(2)
	α (deg)		90	90	90	90	90	90	90	90	90	90
	β (deg)		99.3757	94.6594	96.5083	111.886	98.5498	98.0748	100.605	100.292	100.030(5)	98.2088(8)
	γ (deg)		90	90	90	90	90	90	90	90	90	90
	unit cell vol. (Å^3^)		3121.71	4009.37	3506.68	2684.64	3380.95	3375.63	3236.00	3435.74	3407(2)	3526.56(13)
bond angles (deg)	axial	avg	148.6	151.2	150.2	144.2	149.5	153.3	151.3	150.9	155.8^c^	d
		RMSE	7.7	4.7	5.6	14.8	7.7	7.5	5.6	4.9	d	d
	equatorial	avg	155	157.2	157	152.2	156.0	159.2	158.0	158.0	161.4	d
		RMSE	6.9	4.4	4.7	12.7	6.7	6.5	4.7	4.0	d	d
bond lengths (Å)	axial (external)	avg	3.148	3.245	3.177	3.141	3.212	3.227	3.189	3.227	3.150	d
		RMSE	0.002	0.095	0.027	0.009	0.062	0.077	0.039	0.077	d	d
	axial (internal)	avg	3.178	3.288	3.215	3.19	3.277	3.236	3.172	3.287	3.257	d
		RMSE	0.079	0.031	0.042	0.067	0.020	0.021	0.085	0.030	d	d
	equatorial	avg	3.115	3.251	3.173	3.094	3.184	3.163	3.125	3.190	3.167	d
		RMSE	0.054	0.084	0.013	0.100	0.025	0.009	0.043	0.024	d	d
octahedral tilt (deg)	tilt in (110) plane		4.04	3.56	1.96	2.62	3.16	2.02	1.22	0.57	0.54	d
	rotation about [001]		31.04	28.62	29.64	35.61	30.51	26.57	28.14	28.93	23.97	d
interplane distance (Å)	Pb–Pb across bilayer		15.37	18.71	16.89	13.22	15.88	15.95	15.75	15.95	15.71	d
	ligand N–perovksite I		0.70	0.58	0.64	0.08	0.63	0.71	0.69	0.64	0.75	d

a Measured at 100 K, see ref (18).

b Measured at 298 K, see ref (37).

c No standard deviation reported; data taken from crystallographic.cif file.

d Not reported

Qualitatively, the relaxed geometries are in agreement with experimental structures. We find that ligands in the BA-based system extend perpendicularly from the perovskite subphase, whereas ligands in the HA-based system extend out diagonally with greater “interlocking” of the aliphatic heads. We also find that out-of-phase octahedral tilting is observed along [110] in the BA-based system, compared to along [001] in the HA-based system.

#### Comparison of Exchange-Correlation Functionals

Quantitatively, however, different exchange-correlation functionals lead to large variations in structural features. Without dispersion correction schemes, LDA and PBE produce significantly over- and under-bound structures, respectively. PBEsol gives intermediate results between those of LDA and PBE, which are much closer to experimental lattice constants. Applying simple dispersion correction schemes in the form of pairwise attractive potentials binds the system further. While this leads to large discrepancies in the case of LDA, it significantly improves the lattice constants for relaxed structures at the PBE level. Among the GGA with dispersion results, we find that PBE+G06 significantly overestimates the octahedral tilt between adjacent lead-iodide octahedra in both the BA- and HA-based perovskites, when compared to PBE+TS and PBEsol+TS.

In general, PBE+TS and PBEsol+TS give similar results across most benchmarked structural features. Both give better estimates for the thickness of the ligand bilayer (see Pb–Pb distance across bilayer) compared to PBEsol without any dispersion corrections. PBEsol+TS fares slightly better in describing the inorganic–organic interface— in both systems, PBEsol+TS more accurately reproduces experimental bond lengths for externally facing Pb–I bonds (i.e., those pointing toward the ligand bilayer), as well as the average overlap between the ligand and perovskite (see interplane distance betwen ligand N and perovskite I atoms). However, PBE+TS fares slightly better for the internal axial and equatorial Pb–I bond lengths. Compared to the pairwise dispersion correction schemes, we did not find that the many-body dispersion correction scheme by refs (32) and (33) gives structures significantly closer to the experimentally reported ones. We conclude that there is no single most accurate method; PBE+TS, PBEsol+TS and PBE+MBD all provide reasonable estimates for the equilibrium structure of the two layered perovskites. One may select a method based on which structural features are most important for their work and the availability of computational resources. We note that even the most accurate DFT method benchmarked in this study, PBE+MBD, shows some discrepancies when compared to the experimental reference values. We discuss these in the Supporting Information. We use PBEsol+TS for our calculations of vibrational properties.

#### Comparison of Molecular Dipole Orientations

Having determined a suitable level of theory to study layered perovskites, we compare lattice parameters and structural features obtained for the MA-based perovskites with different MA configurations. We proceed only with monoclinic (HA)_2_(MA)Pb_2_I_7_ because, upon removing symmetry constraints during the geometry optimization, the orthorhombic (BA)_2_(MA)Pb_2_I_7_ structure relaxes to a pseudomonoclinic structure. This is likely unphysical, since experimental studies^19,38^ report a transition directly from the low-temperature triclinic phase to the orthorhombic phase at 283 K.

Predicted lattice constants for (HA)_2_(MA)Pb_2_I_7_ are presented in the Supporting Information. We focus our discussion on the orientations of the MA cations before and after geometry relaxation. This is summarized in Table 3. Consistently across all DFT methods used, we find that configuration 2, which is initialized with MA dipoles pointing along the *c*-axis, relaxes to a final structure with MA dipoles lying in the *ab*-plane. All other configurations, which are initialized with MA dipoles lying in-plane, remain so. Further, within the perovskite plane, neighboring MA dipoles can have antiparallel, parallel, or crossed alignment, with energetic differences on the order of 20 meV per primitive cell (<0.2 meV per atom). We note that these energetic differences may not be solely due to the MA dipole alignnment, since slight changes in the HA ligand geometries are associated with a very flat potential energy surface.

**Table 3:**
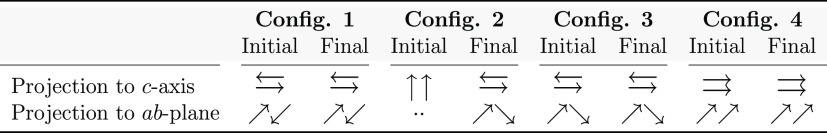
Comparison of the Initial and Final Orientations for MA Configurations Considered

Overall, our calculations indicate a strong orientational preference for MA dipoles to lie *within*, rather than *orthogonal to*, the two-dimensional perovskite layer, at least at temperatures below the energy barrier for MA rotational motion. Further, given the small energetic differences between the various in-plane configurations, (HA)_2_(MA)Pb_2_I_7_ likely has relatively disordered MA dipole orientations throughout the crystal. Prior experimental studies on 3D perovskites^39−41^ have reported that alignment of the molecular C–N bond along the *c*-axis is disfavored in low-temperature phases.

The issue of whether MA cations align macroscopically to form polar domains in 3D perovskites is highly contested.^42−46^ Among quasi-2D perovskites, ferroelectricity has been reported in a propylammonium/methylammonium-based system^47^ and in a butylammonium/methylammonium system.^8^ For our hexylammonium/methylammonium system, ref (19). and ref (18). reported a centrosymmetric monoclinic structure (*C*2/*c* space group) while ref (37). reported a noncentrosymmetric structure (*Cc* space group). Our calculations suggest ferroelectricity arising from permanent, macroscopic polarization of MA cations along the *c*-axis is unlikely in the hexylammonium/methylammonium system. We contrast our results with those of ref (8)., who proposed that the orthorhombic phase of the butylammonium/methylammonium perovskite crystallized in a noncentrosymmetric space group, with MA dipoles aligned with the *c*-axis, resulting in an unquenched dipole moment and ferroelectric behavior.

### Vibrational Properties

#### Zone-Center Modes

We begin with a summary of the Γ-point phonon modes for (HA)_2_CsPb_2_I_7_, which contains hexylammonium (HA) as organic ligands and cesium (Cs) at the A-site cations. The primitive cell of this system has 112 atoms, yielding 336 normal modes. We consider 100 of these modes to be in the low energy regime, that is, below 25 meV (200 cm^–1^). We focus our analysis on these low energy modes, since they are more likely to be appreciably occupied at room temperature (*k*_B_*T* ≈ 26 meV at 298 K). In fact, all Γ-point phonon modes above 15 meV (above mode 86) have <5% contribution from the inorganic (perovskite) subphase. Since band edges in the electronic structure, and therefore, most properties of interest, derive from orbitals of atoms in the inorganic subphase, this further motivates our attention to phonon modes involving predominantly the inorganic subphase.

**Figure 2:**
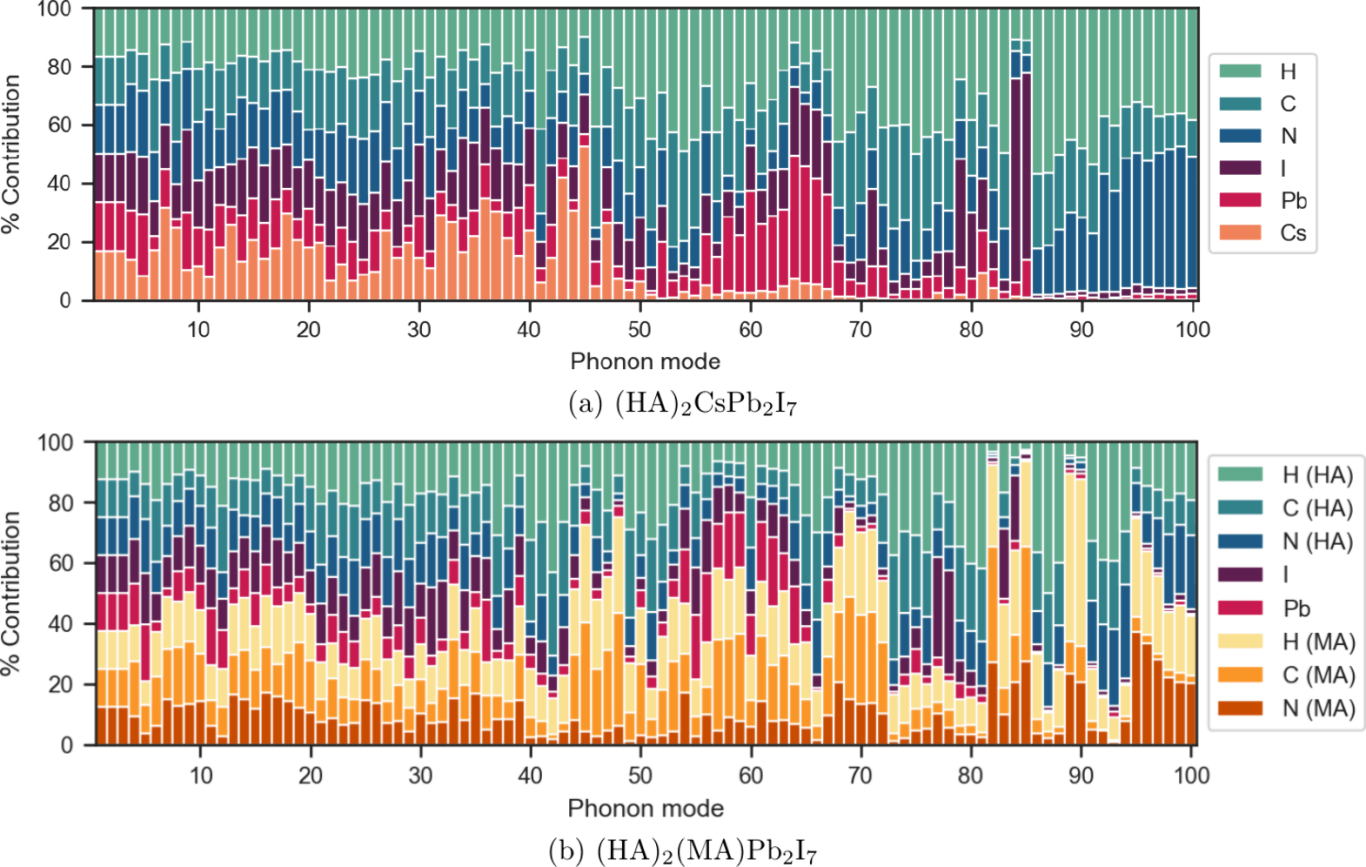
Atom-resolved contributions for the first 100 Γ-point phonon modes (<25 meV) of (HA)_2_CsPb_2_I_7_ and (HA)_2_(MA)Pb_2_I_7_. Note: relative contributions are scaled by the number of atoms in the system.

The partial contribution of each atomic species to the first hundred Γ-point phonons is shown in Figure 2a. The first three modes are the acoustic branches, corresponding to rigid translations with equal contribution from each species. Modes 4 through 40 (1.3–6.5 meV) consist of mixed modes with contributions from the inorganic perovskite lattice, the A-site cation (Cs), and the organic ligand bilayer. In this region, the ligands participate as rigid bodies being “pulled” along by motion of the heavier perovskite lattice. This is reflected in the relative contributions of C, H and N atoms to each mode, which are roughly equal. Modes 41 (6.6 meV) and 48–55 (8.4–9.9 meV) predominantly arise from vibrations of the ligand bilayer. Modes 42–47 (6.8–8.2 meV) have a particularly high contribution from Cs compared to other modes. Modes 56–67 (10.1–11.1 meV) predominantly arise from the perovskite lattice, specifically from displacements of Pb atoms at the center of the lead-iodide octahedra. Modes 84 (14.7 meV) and 85 (14.9 meV) exhibit high contributions from the I atoms of the inorganic subphase. From mode 86 (15.9 meV) onward, contributions from the inorganic subphase vanish, leaving only vibrations of the ligand bilayer.

In general, the motions of the inorganic subphase within (HA)_2_CsPb_2_I_7_ bear resemblance to known vibrational modes of the 3D perovskite structure. We identify modes corresponding to stretching of Pb–I bonds, bending and rocking of I–Pb–I bonds, off-center displacement of the central Pb in lead-iodide octahedra, tilts and rotations of lead-iodide octahedra, and rattling of the A-site cation inside the lead-halide cage. Detailed examples are given in the Supporting Information.

**Figure 3:**
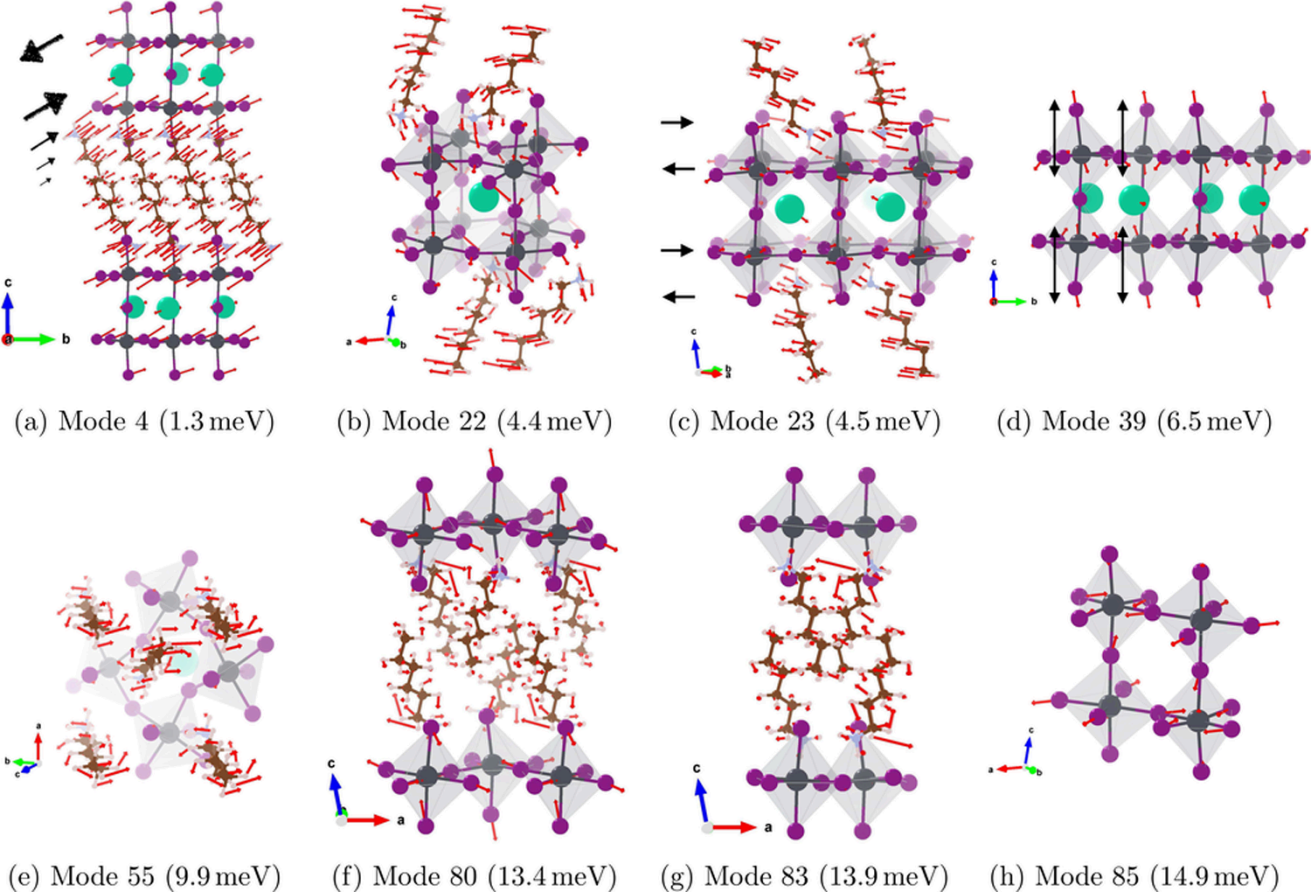
Eigendisplacements of coupled organic–inorganic phonon modes and of phonon modes unique to the layered perovskite (i.e., not found in the bulk perovskite and ligand reference systems) discussed in the main text.

Interestingly, we also observe vibrations of the inorganic subphase that have no counterpart in the 3D perovskite structure. Examples include mode 4 (Figure 3a), where the entire perovskite subphase undergoes a shearing motion which is dampened by the soft ligands; mode 39 (Figure 3d), where the entire perovskite subphase expands and contracts vertically along the *c*-axis; and mode 85 (Figure 3h), where the classic perovskite octahedral breathing mode includes 5, rather than all 6, Pb–I bonds. Modes 4 and 39 are only possible because the dispersion forces binding the organic bilayer are relatively weak, allowing the ligands to slide past each other in the center and effectively “dampen” the disruption to the lattice. By contrast, in a 3D crystal, such vibrations of the perovskite sublattice would deform the entire crystal structure. Mode 85 is analogous to the octahedral breathing mode at 13.9 meV (111.56 cm^–1^) in tetragonal MAPbI_3_.^48^ However, because the external Pb–I bonds interfacing with the organic bilayer experience a different local environment, they do not vibrate together with the internal Pb–I bonds.

Like the inorganic lattice, vibrations of the organic subphase in (HA)_2_CsPb_2_I_7_ often derive from phonon modes of the free molecule and/or the molecular crystal. However, the exact frequencies of those vibrational modes, such as ammonium bending, are modified by the perovskite environment, similar to findings of Lavan et al.^49^ We provide a more extensive discussion of phonons for the free molecule and molecular crystal in the Supporting Information. Nevertheless, like the perovskite subphase, the organic subphase also exhibits modes that correspond to *neither* reference system. One example involves localized motion in one segment of the HA molecule, as in mode 83 (Figure 3g). For our discussion, we define C1 as the α carbon immediately adjacent to the ammonium group and C6 as the last carbon of the alkyl tail. In mode 83, the C2–C3 bond undergoes torsion and “swings” the entire CH_2_ group at C1 around the molecule’s central N–C6 axis. We did not observe this internal mode in the free molecule nor molecular crystal, so it likely emerges because the HA ammonium group interfacing with the perovskite subphase experiences a different local environment. Another is mode 55 (Figure 3e), which consists of rotations of all CH_2_ moieties about the C–C axis in the *same* direction, similar to one of the rotational degrees of freedom for a free HA molecule. We attribute this mode to the larger intermolecular spacing in the layered perovskite system compared to the molecular crystal. The nearest neighbors for a given HA ligand in the layered perovskite are 5.3 Å and 6.7 Å away (defined by distance between the N atoms), compared to 3.7 Å and 4.8 Å away in the molecular crystal. Thus, the ligand bilayer exhibits phonon modes akin to independently rotating HA molecules, even in the absence of surrounding lead-iodide octahedra rotations. Finally, we find that molecular vibrational frequencies, such as ammonium bending, are modified by the perovskite environment, similar to findings of Lavan et al.^49^

Having summarized the vibrations of the perovskite- and organic- subphases, we now discuss how they couple to each other. For the first ∼40 modes up to 6.5 meV, the organic subphase participates in phonon modes as a rigid body being “pulled along” by vibrations of the much heavier perovskite subphase. This is illustrated in Figure 3c, where HA ligands follow the direction of motion of the *external*-facing I atoms, i.e., the I atoms sticking out at the interface. Our characterization is similar to that of ref (16), though we find that coherence of the ligand motion extends all the way to the sixth carbon, while they found that coherence of the ligand motion extends only up to the fourth carbon. Further, for phonon modes involving predominantly rotations of lead-iodide octahedra about the *c*-axis, that rotational motion also manifests in the organic subphase. For example, Figure 3c illustrates the vine-like twisting motion around the *c*-axis in the same direction of rotation as surrounding lead-iodide octahedra.

At higher energies, we find many modes that appear to be a mix of the perovskite-dominated and ligand-dominated modes discussed above. One example is mode 80 (Figure 3f). Here, the HA ligands exhibit torsion about the C2–C3 bond that swings the C1 methyl group around, akin to mode 83 (Figure 3g) discussed earlier. At the same time, the perovskite subphase exhibits stretching and contracting of equatorial and axial Pb–I bonds inherited from similar energy breathing modes of the lead-iodide framework in 3D perovskites. This result suggests the ligand and perovskite subphases in this energy regime vibrate independently, with mixed modes arising from linear combinations of perovskite-dominated and ligand-dominated normal modes.

#### IR Spectra

**Figure 4:**
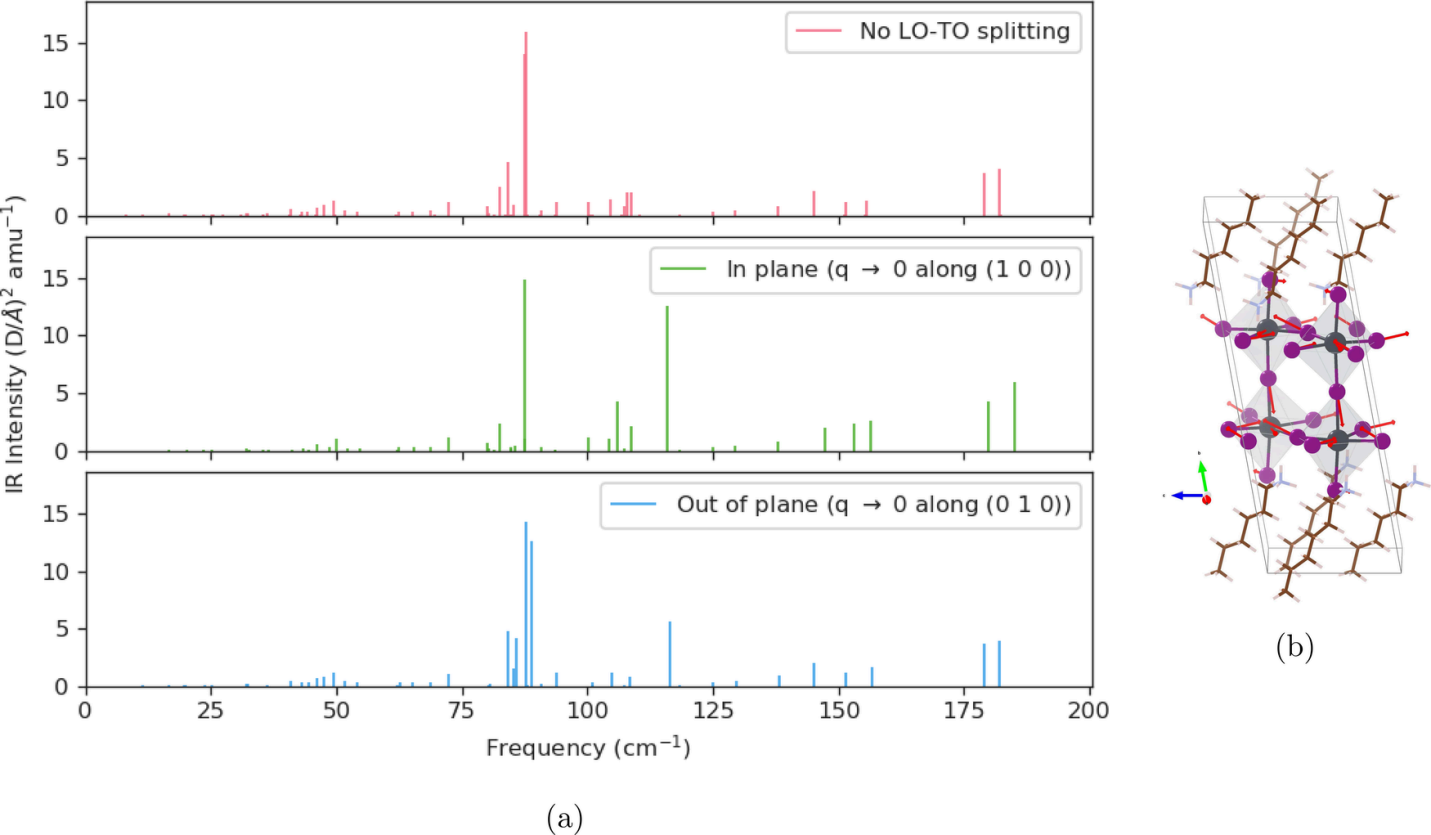
(a) Predicted IR spectra for (HA)_2_CsPb_2_I_7_ in the low-frequency regime. (b) Eigenvectors of the normal mode with large LO-TO splitting.

We additionally show the predicted IR spectra for low-frequency vibrational modes in (HA)_2_CsPb_2_I_7_ (Figure 4). The IR spectra over higher frequencies, which all correspond to vibrational modes of organic molecules, are given in the Supporting Information. We additionally compute the nonanalytic correction to the vibrational frequencies in the limit where *q* → 0 for both the in-plane and out-of-plane directions. We find small LO-TO splitting for most normal modes, with the exception of one mode at 87 cm^–1^, which exhibits 30 cm^–1^ LO-TO splitting. This mode, which involves stretching of the Pb–I network, is qualitatively similar to the large LO-TO splitting mode of *B*_2*u*_ symmetry identified by Pérez-Osorio et al.^50^ in their study of MAPbI_3_, showing that quasi-2D perovskites inherit the vibrational properties of its constituent phases. We note that our computed frequency for this mode is 20 cm^–1^ higher than theirs, which we again attribute to the fact that I atoms at the perovskite/ligand interface experience a local environment change.

#### Phonon Dispersion

**Figure 5:**
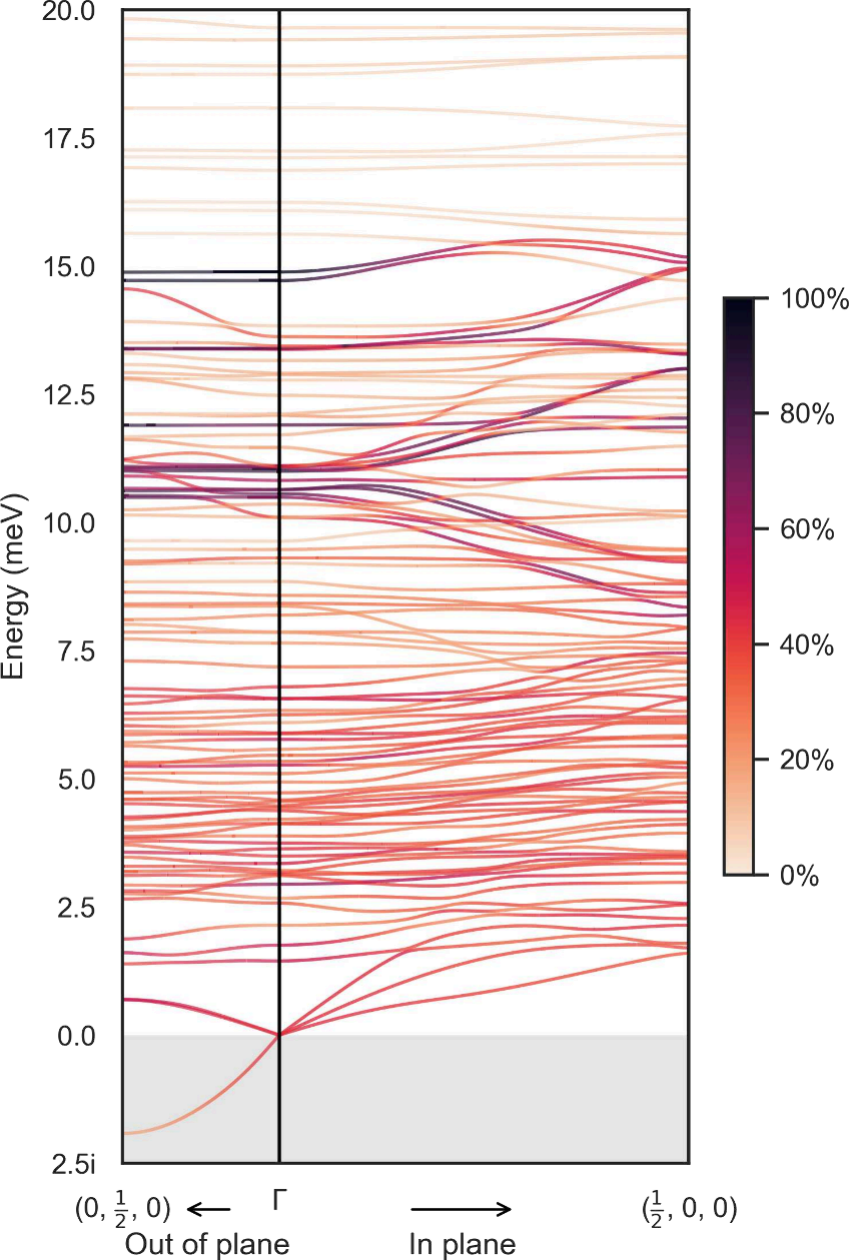
Projected dispersion to the lead-iodide subphase for the low energy region of (HA)_2_CsPb_2_I_7_.

Having characterized the Γ-point phonons in detail, we now turn our attention to the phonon dispersion in the in-plane and out-of-plane directions. The low energy region of the phonon dispersion is presented in Figure 5. There is a slightly imaginary frequency associated with the longitudinal acoustic mode in the out-of-plane direction, which we attribute to numerical noise confirmed through frozen-phonon calculations (see Supporting Information). We additionally project the phonon eigenvectors along the in-plane and out-of-plane paths to the three subsystems of the layered perovskite: the lead-iodide framework, the ligand bilayer, and the A-site cation. The projection to the lead-iodide framework is given as the colors in Figure 5 while the others are presented in the Supporting Information.

While the dispersion is highly complex, we can draw a few general conclusions. First, most phonon branches are dispersionless. Second, for the subset of branches that do show some dispersion, they are limited to modes below 15 meV, i.e. the ultra low energy modes. Third, by projecting the phonon eigenvectors onto the different subphases of the system, we find that the dispersive branches are dominated by vibrations of the lead-iodide framework in the perovskite subphase. Given that the electronic band edges arise from the inorganic lattice, these dispersive phonon modes involving the inorganic lattice are excellent candidates for future investigations into carrier-phonon coupling in quasi-2D perovskite systems. This is because spatial overlap of the electronic and vibrational excitations primarily determine coupling.^51,52^ For example, similar modes, involving I–Pb–I stretching and I–Pb–I bending, have been observed to form electron-polaron states in bulk MAPbI_3_.^53^ Longitudinal optical phonons involving stretching of the Pb–I equatorial bond network around 10–13 meV (similar to the branches around 10–11 meV at Γ) were also identified as the origin of PL spectra broadening in MAPbBr_3_.^54^

Finally, the dispersion is highly anisotropic. Those perovskite subphase branches are much more dispersive in the in-plane direction than the out-of-plane direction. For example, the branches that start at around 10–11 meV at Γ and end at around 8–9 and 12–13 meV at  are associated with bending and breathing modes of the equatorial Pb–I network, i.e., Pb–I bond stretching coupled to Pb displacing to an off-center position. On the other hand, the branch that starts around 12.0 meV at Γ and remains flat to  is associated with axial displacements of the internal I atoms. In other words, the in-plane phonons (i.e., those involving equatorial motion) are more dispersive than out-of-plane phonons (i.e., those involving axial motion) along the path from Γ to either zone boundary. This is consistent with the two-dimensional nature of the perovskite subphase. However, we note that we did not observe a quadratic dispersion relation for the out-of-plane acoustic branch characteristic of 2D materials.^55^ This suggests that layered perovskites have some two-dimensional nature but are distinct from pure 2D materials such as graphene. One final interesting feature in the phonon dispersion is the lack of a gap between the longitudinal acoustic branch and the lowest energy optical branch. This is an inherited property from 3D perovskite systems, where gaplessness has been hypothesized to be responsible for short phonon lifetimes and low thermal conductivity.^56,57^ The complexity of the phonon dispersion certainly merits further investigation; nevertheless, the results presented here provide a valuable foundation for studying how phonons can interact with charge carriers and other phenomena in layered perovskite systems.

#### Comparison of Cs-Based and MA-Based Perovskites

**Figure 6:**
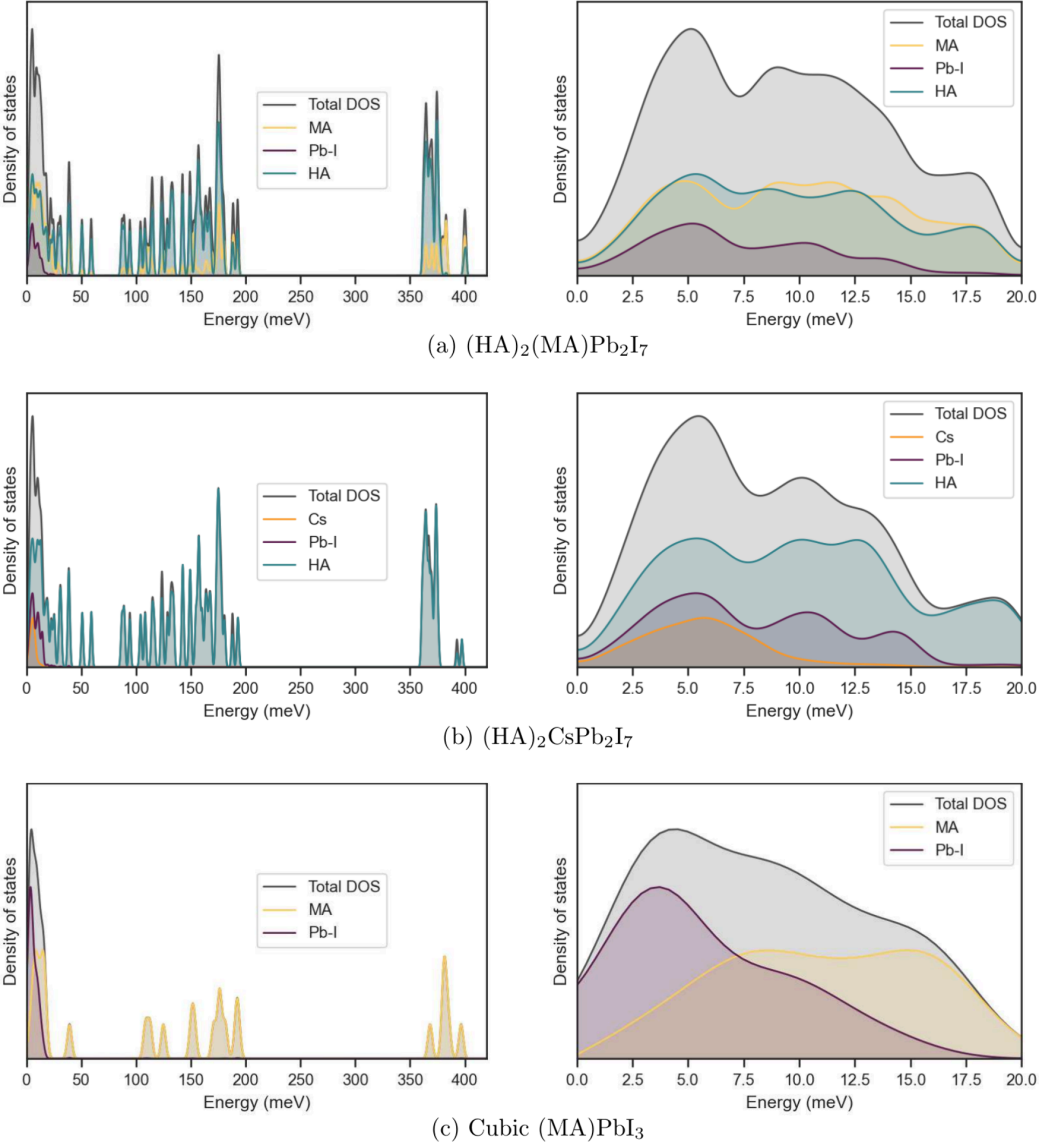
Total and subsystem-resolved vibrational density of states for (HA)_2_(MA)Pb_2_I_7_, (HA)_2_CsPb_2_I_7_, and (MA)PbI_3_. Data for (MA)PbI_3_ are reproduced from ref (48).

We turn our discussion to vibrational properties of the MA-based layered perovskite. Figure 2b presents the atom-resolved partial contributions to the first 100 Γ-point phonons and Figure 6 presents the atom-resolved density of states.

Immediately, we observe that the light methylammonium cation occupying the A-site couples to the vibrations of the lead-iodide lattice across all of the low energy regime. By contrast, the heavier Cs cation in the Cs-based perovskite does not show significant contribution past 10 meV. Below 7.5 meV (mode 40), the motion of the methylammonium cation is best described as a rigid-body translation, similar to the “rattling” of Cs cations in the lead-iodide cage discussed earlier. This is evidenced by the relatively equal weights of its constituent atoms: H, C, and N. Above 7.5 meV, there are numerous modes where the relative contribution of C and H is much greater than N. These correspond to librations of the MA cation, where the ammonium group is fixed (presumably due to strong interactions with the lead-iodide lattice) while the methyl group moves. The observed frequency for this type of MA libration in our layered perovskite system is consistent with studies on MAPbI_3_ by refs (48, 50, and 58), where it has also been described as a “nodding donkey” vibrational mode.

Aside from this main difference, most phonon modes of the Cs-based and MA-based quasi-2D perovskites resemble each other and are largely independent of the A-site cation. In both perovskite systems, we find octahedral distortion, rotational, and breathing modes in the perovskite subphase at similar frequencies. We also observe similar bending and twisting modes of the ligand backbone around 7.5–7.7 meV and 9.1–9.8 meV. Thus, we expect dispersion behavior in the Cs-based and MA-based quasi-2D perovskites to be similar. We note that the dispersive, low-energy, perovskite-dominated optical modes characterized in this work have been shown to play a role in exciton-polaron formation by prior studies. Reference (14) observed that different excitons coupled to different low-frequency optical phonons of the perovskite subphase, which were signatures of polaron formation. However, whether or not the organic ligand subphase has an impact on exciton-polaron formation remains an open question. Reference (59) reported that the choice of the organic cation determined to which vibrational modes the exciton couples. Specifically, excitons coupled to a phonon mode at 100 cm^–1^ in *n* = 1 butylammonium (BA) lead iodide but to phonon modes at 88 and 137 cm^–1^ in *n* = 1 hexylammonium (HA) lead iodide. On the other hand, work by ref (16) on a series of *n* = 1 alkylammonium lead iodide perovskites ranging from four to nine carbons showed no change in exciton–phonon coupling, which suggests that the exciton is highly confined to the inorganic subphase and that polaron formation is dominated by the motion of the inorganic subphase. We emphasize that the perovskites used in refs (59) and (16) are distinct to those studied in this work because ours are *n* = 2 layered perovskites. We hope that the detailed characterization of phonon modes described in this work will provide a foundation for future investigations of exciton–phonon coupling in quasi-2D perovskites, such as potential mechanisms for how phonons may modulate exciton spin dynamics.^13,60^

## Conclusion

In summary, we present a comprehensive study of phonon properties from first-principles for quasi-2D perovskites (BA)_2_CsPb_2_I_7_, (HA)_2_CsPb_2_I_7_, (BA)_2_(MA)Pb_2_I_7_, and (HA)_2_(MA)Pb_2_I_7_. We first benchmark DFT levels of theory to accurately describe their structural properties, concluding that semiempirical dispersion corrections improve the agreement between the computed and experimental equilibrium structure. We then investigate preferred alignments of the MA molecular dipoles in the ground state structures. Our results indicate that macroscopic alignment of MA dipoles in the out-of-plane direction is disfavored and support assignment of the centrosymmetric space group for the low-temperature, monoclinic phase of (HA)_2_(MA)Pb_2_I_7_. We finally compute vibrational properties for (HA)_2_CsPb_2_I_7_ and (HA)_2_(MA)Pb_2_I_7_. Our results suggest that phonons in quasi-2D perovskites are largely inherited from their constituent parts, with the inorganic (perovskite) and organic (ligand bilayer) subphases of quasi-2D peroskite vibrating mostly independently. However, there are some unique, coupled modes that would not be observed in bulk 3D perovskites or ligand molecular crystals. Moreover, among the low-energy phonon branches, we find much steeper dispersion in the in-plane direction (i.e with the perovskite subphase) compared to the out-of-plane direction. The most dispersive branches are related to bending and breathing modes of the equatorial Pb–I network within the perovskite plane.

## Data Availability

Github Repository: Input files for DFT calculations and data files (frequencies and eigenvectors) for all phonon modes discussed in this manuscript (https://github.com/emilyyanchen/quasi-2d-perovskite-phonons).

## Supporting Information Available

Detailed computational methodology, convergence tests, structural data for quasi-2D perovskites not shown in the main text, additional views of crystal structures for quasi-2D perovskites, an in-depth discussion of all low energy vibrational modes (PDF).
