# Supplementary material for: Lattice Dynamics of Quasi-2D Perovskites from First Principles
Source: J Phys Chem C Nanomater Interfaces. 2024 Jul 16;128(29):12194–205. doi: 10.1021/acs.jpcc.4c01633 (PMC11284783; doi:10.1021/acs.jpcc.4c01633)
Supplement: Supplementary file 1 — jp4c01633_si_001.pdf [file jp4c01633_si_001.pdf]

Supporting Information: Lattice dynamics of Quasi-2D Perovskites from First-Principles

Emily Y. Chen*,†,‡ and Bartomeu Monserrat*,†,¶,§

†*Cavendish Laboratory, University of Cambridge, J. J. Thomson Avenue, Cambridge CB3 0HE, United Kingdom*

‡*Department of Materials Science and Engineering, Stanford University, 496 Lomita Mall, Stanford, CA 94305, U.S.A.*

¶*Department of Materials Science and Metallurgy, University of Cambridge, 27 Charles Babbage Road, Cambridge CB3 0FS, United Kingdom*

§*Corresponding author*

E-mail: cemily@stanford.edu; bm418@cam.ac.uk

We provide structural data, phonon frequencies and phonon eigenvectors discussed in this paper https://github.com/emilyyanchen/quasi-2d-perovskite-phonons.git.

Detailed methodology

As mentioned in the main text, we obtain crystallographic data for $(BA)_2(MA)Pb_2I_7$ from Ref. 1 and for $(HA)_2(MA)Pb_2I_7$ from Ref. 2. We generate primitive cells using the `cif2cell`[3] tool and an example is shown in Figure S2a. We also construct structural models where the MA molecules are replaced with Cs atoms, i.e. $(BA)_2CsPb_2I_7$ and $(HA)_2CsPb_2I_7$, a common procedure in theoretical calculations of such perovskites to reduce the computational complexity associated with the rotational disorder of MA cations.[4] Cs and MA have similar

ionic radii of 1.74 Å[5] and 1.80 Å,[6] respectively. Neither contribute significantly to the electronic structure near the valence and conduction bands, which are primarily responsible for bonding. As such, the spherically symmetric Cs can be considered a proxy for the average over many MA orientations.

Table S1: Overview of the four systems considered in this report

**Compound**	**$(BA)_2(MA)Pb_2I_7$**	**$(BA)_2(Cs)Pb_2I_7$**	**$(HA)_2(MA)Pb_2I_7$**	**$(HA)_2(Cs)Pb_2I_7$**
Organic ligand	$C_4H_9NH_3$	$C_4H_9NH_3$	$C_6H_{13}NH_3$	$C_6H_{13}NH_3$
A-site cation	CH_3NH_3	Cs	CH_3NH_3	Cs
Num. perovskite layers	$n = 2$	$n = 2$	$n = 2$	$n = 2$
Crystal system at 298 K	orthorhombic	orthorhombic	monoclinic	monoclinic
Space group	$Cc2m$ or $Ccmm$	$Ccmm$	C2/c or Cc	C2/c

A summary of the systems of interest is shown in Table S1. The difference between the two orthorhombic ($Cc2m$ and $Ccmm$) and monoclinic (C2/c or Cc) space groups refers to whether the MA cations are oriented such that inversion symmetry is present. We note that, in real systems, MA cations may be oriented differently in different unit cells, leading to local distortions in the crystal structure. Due to computational constraints, we can not construct large supercells with stochastically distributed MA orientations. We therefore enumerate four configurations meant to represent the "extremes" of the possible MA dipole orientations, described in the main text. In particular, we distinguish the configurations by (i) whether the central C−N bond of the MA molecule lies flat within the perovskite layer or is orthogonal to the perovskite layer; (ii) whether the molecular dipoles of the two MA molecules per unit cell are antiparallel (180°), parallel (0°), or crossed (90°). The three possible in-plane alignments are illustrated in Figure S1.

We perform electronic structure calculations within the Kohn-Sham density functional theory (DFT) framework[7,8] in `CASTEP`[9] using ultrasoft pseudopotentials.[10] We use the Cs-based perovskite models to benchmark the performance of three different exchange-correlation functionals, namely the Perdew-Zunger local density approximation (LDA),[8,11,12] the Perdew–Burke–Ernzerhof (PBE) generalized gradient approximation functional,[13] and the modified Perdew–Burke–Ernzerhof generalized gradient approximation functional for

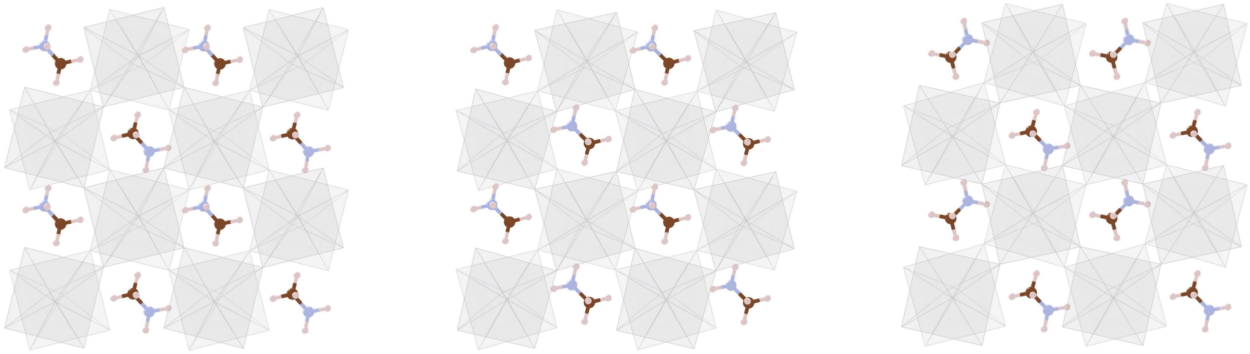

(a) Anti-parallel (b) Parallel (c) Crossed

Figure S1: Possible in-plane alignments of MA dipoles

solids (PBEsol).[14] We additionally test four semi-empirical dispersion correction schemes, namely those by (i) Ortmann, Bechstedt, and Schmidt[15] (OBS) (ii) Grimme[16] (G06) (iii) Tkatchenko and Scheffler[17] (TS) and (iv) a many-body scheme by Tkatchenko and co-workers[18,19] (MBD). We do not include spin-orbit coupling effects, which have been shown to primarily effect electronic properties (e.g. the band gap) but not vibrational properties (e.g. phonon frequencies and dispersion) in other halide perovskites.[20,21]

For all geometry optimization calculations, we use a kinetic energy cutoff of 600 eV for the plane wave basis set and a spacing of $2\pi \times 0.04\,\text{Å}^{-1}$ between **k**-points for the Γ-centered Monkhorst-Pack mesh for Brillouin zone sampling (corresponding to a $3 \times 2 \times 3$ grid). For the Cs-based structural models, we constrain the symmetry (but not the cell dimensions) based on the known experimental crystal structures. For the MA-based structural models, since our choice of MA orientation necessitates breaking the known crystal symmetry, we do not apply any constraints. Convergence is reached when (i) all lattice constants and angles are converged within 0.01 Å and 0.01 deg, (ii) the maximum force on any ion is less than 0.01 eV/Å, and (iii) components of the stress tensor are less than 0.1 GPa. Where a semi-empirical dispersion correction is used, the converged structure is used as input for a few more geometry optimization steps. This ensures convergence, since for some correction schemes, the correction parameters are determined by the initial structure and not the final structure. No modifications are made to `CASTEP` default parameters for dispersion corrections. We

compare the relaxed structures using a few descriptors, shown in Figure S2.

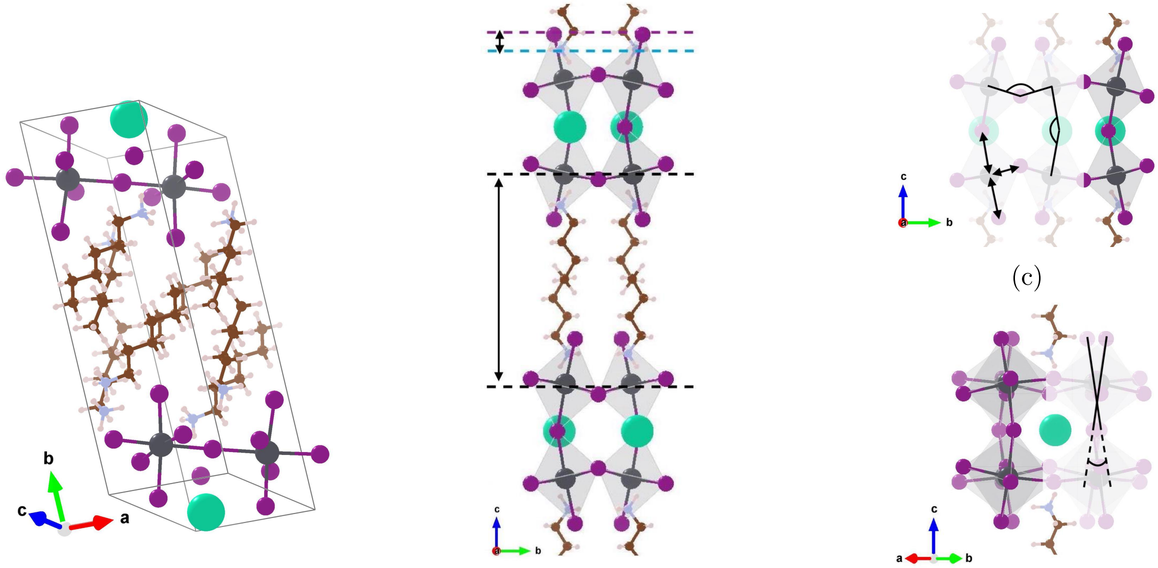

(a) (b) (c) (d)

Figure S2: (a) Primitive cell for $(HA)_2(Cs)Pb_2I_7$; (b) Schematic showing the ligand N/perovskite I overlap distance (top) and the Pb–Pb interplane distance across the bilayer (bottom); (c) Schematic showing differences between axial Pb–I bonds and Pb–I–Pb angles, which lie along the c-axis, and equatorial Pb–I bonds and Pb–I–Pb angles, which lie in the a,b-plane on $(BA)_2(Cs)Pb_2I_7$; (d) Schematic showing the octahedral tilt angle between adjacent lead-iodide octahedra. Colours are cyan (Cs), purple (I), grey (Pb), brown (C), pink (N) and white (H)

We then choose $(HA)_2CsPb_2I_7$ as our model system to study vibrational properties. The other systems benchmarked are not suitable for a few reasons: (i) we optimize the high-temperature orthorhombic phase of $(BA)_2CsPb_2I_7$, which would result in imaginary phonon frequencies for implicitly 0 K calculations; (ii) we do not obtain the correct space group for the low-temperature phase of $(BA)_2(MA)Pb_2I_7$ from geometry optimization, and (iii) the MA cations in $(HA)_2(MA)Pb_2I_7$ may be associated with significant anharmonic effects, as they are in 3D perovskites. Such soft phonon modes would be challenging to disentangle with imaginary modes indicating a poorly optimized structure. Thus, we proceed with $(HA)_2CsPb_2I_7$, keeping in mind that this system can be viewed as a proxy for the dynamically averaged crystal structure of $(HA)_2(MA)Pb_2I_7$.

We assume the harmonic approximation and simulate lattice dynamics using the direct method.[22] Based on the results from benchmarking different exchange-correlation function-

als and dispersion correction schemes, we first compute Γ-point phonons for the relaxed structures of $(HA)_2CsPb_2I_7$ at the PBE+TS and PBEsol+TS levels of theory. In this first calculation, we displace each ion by 0.02 bohr in each Cartesian direction to obtain force constants. The electronic structure calculation parameters remain as before: $2\pi \times 0.04\,\text{Å}^{-1}$ Monkhorst-Pack sampling grid for the Brillouin zone and 600 eV as the basis set cut-off. For the PBE+TS structure, we find 10 imaginary phonon modes at the Γ point with eigenvalues ranging from $4.7i$ meV ($40i\,\text{cm}^{-1}$) to $0.4i$ meV ($3.0i\,\text{cm}^{-1}$). For the PBEsol+TS structure, we find 3 imaginary phonon modes at the Γ point with eigenvalues at $13i$ meV ($107i\,\text{cm}^{-1}$), $1.9i$ meV ($15i\,\text{cm}^{-1}$) and $0.96i$ meV ($7.8i\,\text{cm}^{-1}$).

Because the PBEsol+TS calculation has fewer imaginary phonon modes and typically converges in fewer iterations during geometry optimization, we proceed with the PBEsol+TS structure. We displace the structure along the atomic displacement patterns of each imaginary phonon mode and calculate the total energy from DFT at a number of points. This maps out the potential energy well associated with each imaginary phonon mode. We find the lowest energy structure along the quartic double-well potential of phonon mode 2, at 13 meV lower than the initial structure (see Figure S3), and use it as input for a new variable-cell geometry relaxation.

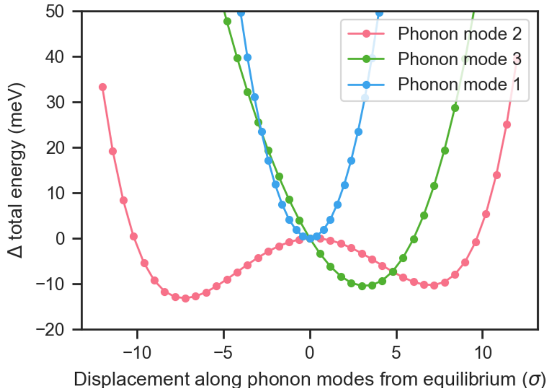

Figure S3: Potential energy landscape along the imaginary phonon modes of the PBEsol+TS equilibrium structure

We repeat the Γ-point phonon calculation for the new structure and find only 1 imaginary

mode. However, the associated potential energy well appears completely harmonic and does not reveal any lower energy structures. Further, the phonon frequency and eigendisplacements of the softest mode varies widely depending on the amplitude of ionic displacement used to compute the force constants. These observations are presented in Figure S4.

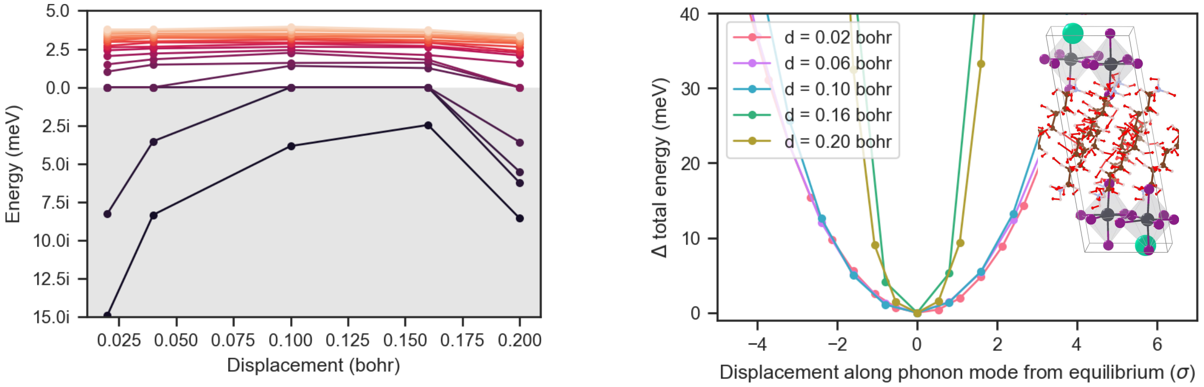

(a) Phonon frequencies of soft modes as a function of displacement amplitude

(b) Potential energy well of the softest mode. Inset: eigenvectors of the softest mode

Figure S4: Phonon frequency, potential energy surface and eigenvectors associated with the persistent soft mode

These puzzling results suggest that numerical noise on the DFT forces is high. We hypothesize that the energy cut-off of 600 eV and convergence criteria of 10^{-7} eV for electronic structure calculations cannot yield sufficiently good quality forces. Even though lattice constants are converged, the positions of individual atoms (especially in the soft organic molecules) likely require more stringent parameters. This is supported by the fact that the phonon eigenvectors of the elusive imaginary mode are randomly oriented and associated with the (very light) hydrogen atoms within the organic ligand layer (see Figure S4b). After additional convergence testing, we increase the basis set cut-off to 800 eV, the convergence tolerance for self-consistent cycles to 10^{-9} eV, and the displacement amplitude to 0.06 Å. These parameters allow us to eliminate the imaginary phonon. We repeat the Γ-point phonon calculation for the MA-based perovskite, $(HA)_2(MA)Pb_2I_7$, using the same parameters as the Cs-based perovskite. Note that this calculation is only for configuration 1, where MA dipoles were antiparallel to each other. We then compute vibrational density of states for

both systems with 10,000 stochastically generated points in the vibrational Brillouin zone. Finally, we report IR spectra, computed using density-functional perturbation theory in `CASTEP`[23] and the same DFT convergence parameters.

We focus on $(HA)_2CsPb_2I_7$ to explore the phonon dispersion. We generate supercells with increased length in the in-plane ($2\times1\times1$) and out-of-plane ($1\times2\times1$) directions. The former probes the two-dimensional nature of the phonons in the perovskite subphase, while the latter probes any inter-layer coupling across the different stacked subphases. We explicitly compute force constants from DFT calculations at the zone-center $(0,0,0)$ and zone boundaries—$(\frac{1}{2},0,0)$ for in-plane and $(0,\frac{1}{2},0)$ for out-of-plane. Force constants for all other points along the given path in the Brillouin zone are obtained via Fourier interpolation. Custom codes are written to interface with the `VESTA`[24] software to visualize phonon eigendisplacements as well as to project phonon eigenvectors to specific atoms and subphases. We define the projection of a given phonon mode ν to subphase λ at wavevector $\mathbf{q}$ as:

$$P_{\lambda,\nu,\mathbf{q}} = \frac{\sum_{\kappa\in\lambda} |\mathbf{e}_\kappa(\mathbf{q},\nu)|^2}{\sum_{\kappa'}^{N} |\mathbf{e}_{\kappa'}(\mathbf{q},\nu)|^2} \tag{1}$$

where $\mathbf{e}_\kappa(\mathbf{q},\nu)$, the vibrational eigenstates for atom κ, comes from the solution to the phonon eigenvalue problem involving the dynamical matrix,

$$[\omega(\mathbf{q},\nu)]^2\,\mathbf{e}(\mathbf{q},\nu) = \mathbf{D}(\mathbf{q})\,\mathbf{e}(\mathbf{q},\nu) \qquad ; \qquad \mathbf{e}(\mathbf{q},\nu) = \begin{pmatrix} \mathbf{e}_1(\mathbf{q},\nu) \\ \mathbf{e}_2(\mathbf{q},\nu) \\ \vdots \\ \mathbf{e}_N(\mathbf{q},\nu) \end{pmatrix} = \begin{pmatrix} e_1^x(\mathbf{q},\nu) \\ e_1^y(\mathbf{q},\nu) \\ e_1^z(\mathbf{q},\nu) \\ \vdots \\ e_N^x(\mathbf{q},\nu) \\ e_N^y(\mathbf{q},\nu) \\ e_N^z(\mathbf{q},\nu) \end{pmatrix} \tag{2}$$

Convergence with respect to basis set size

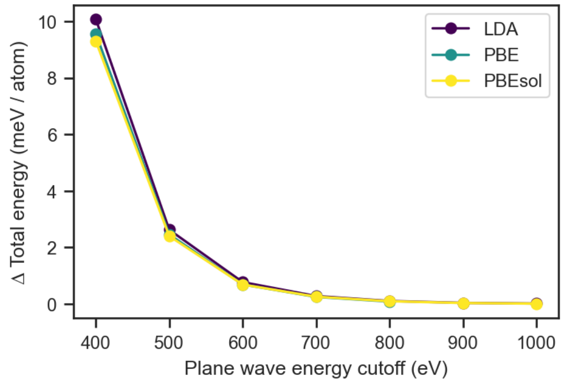

(a) Total energy (unrelaxed)

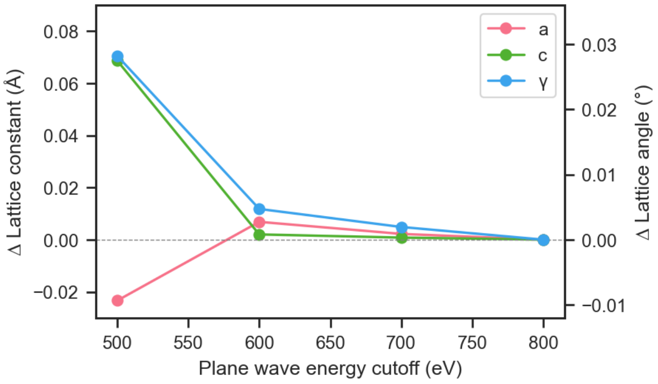

(b) Relaxed lattice constants (PBEsol)

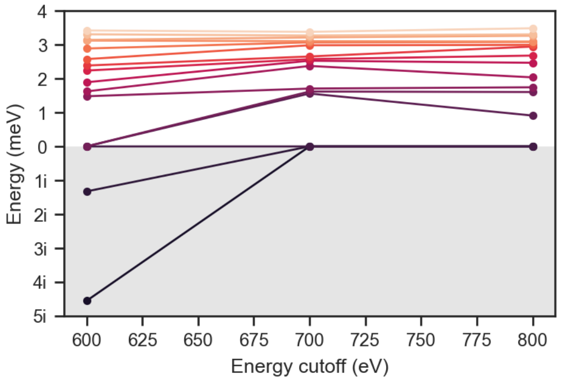

(c) Low energy phonons

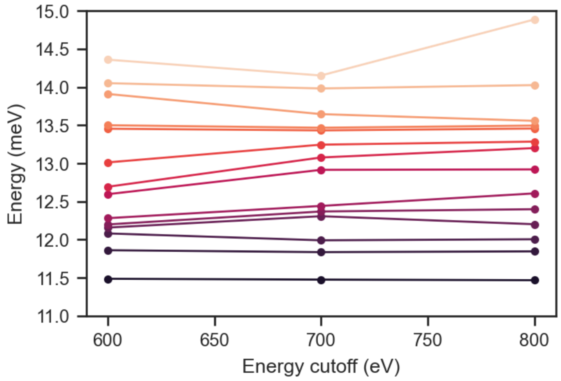

(d) High energy phonons

Figure S5: Convergence with respect to basis set size

Convergence with respect to k-point sampling

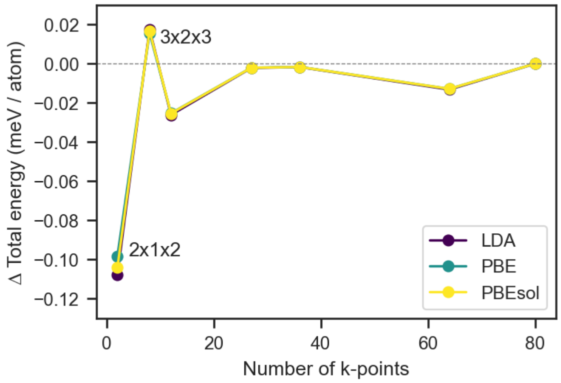

(a) Total energy (unrelaxed)

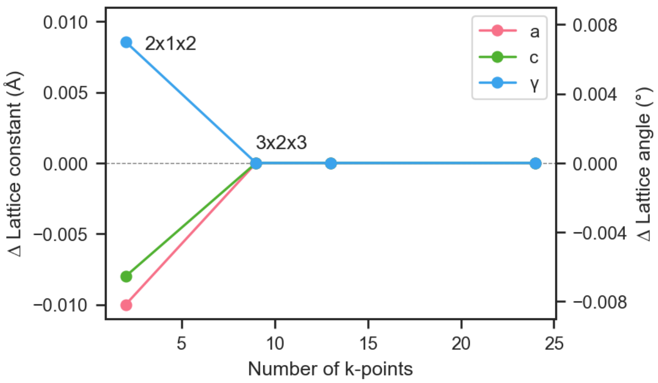

(b) Relaxed lattice constants (PBEsol)

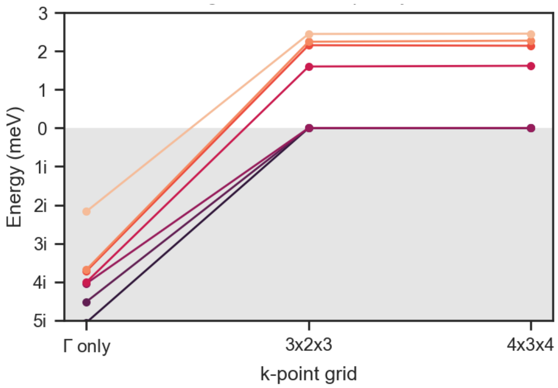

(c) Low energy phonons

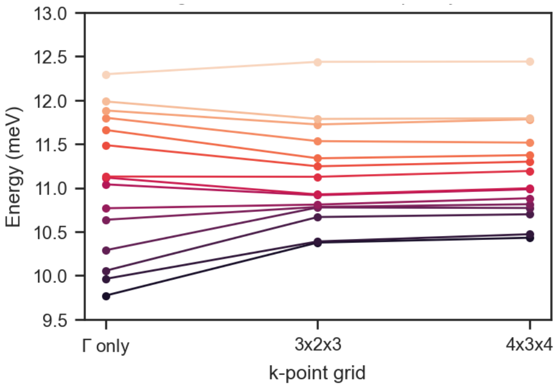

(d) High energy phonons

Figure S6: Convergence with respect to **k**-point mesh

Convergence with respect to displacement amplitude

In the direct method,[22] one obtains force constants by taking numerical derivatives of the forces. We build two structures, one where atom κ' in cell n' is displaced in the β direction by some small amount $+u_\beta$, and the other where that same atom is displaced by $-u_\beta$. For each structure, we compute the resulting force in the α direction on a different atom κ in cell n, which we write as $\mathbf{F}^{\alpha+}_{\kappa,n}$ and $\mathbf{F}^{\alpha-}_{\kappa,n}$. Then, the force constant $\Phi^{\kappa' n' \beta}_{\kappa\, n\, \alpha}$ between atoms κn and $\kappa' n'$, i.e. the derivative of the force at equilibrium, is given by the central difference

$$\Phi^{\kappa' n' \beta}_{\kappa\, n\, \alpha} = \frac{\mathbf{F}^{\alpha+}_{\kappa,n} - \mathbf{F}^{\alpha-}_{\kappa,n}}{2u_\beta} = \left.\frac{\mathbf{F}^{\alpha}_{\kappa,n}}{\partial u^{\beta}_{\kappa',n'}}\right|_{\{\mathbf{r}_{\text{eq}}\}} = -\left.\frac{\partial^2 E}{\partial u^{\alpha}_{\kappa n} \partial u^{\beta}_{\kappa' n'}}\right|_{\{\mathbf{r}_{\text{eq}}\}} \tag{3}$$

Repeating this process for all atom pairs κn and $\kappa' n'$ gives the matrix of force constants, the Fourier transform of the dynamical matrix. Ideally, the displacement amplitude (u_β) should be small enough to remain in the harmonic regime, but not so small that numerical noise on the force constants dominate. For a sufficiently harmonic system, computed phonon frequencies should be largely independent of the chosen displacement.

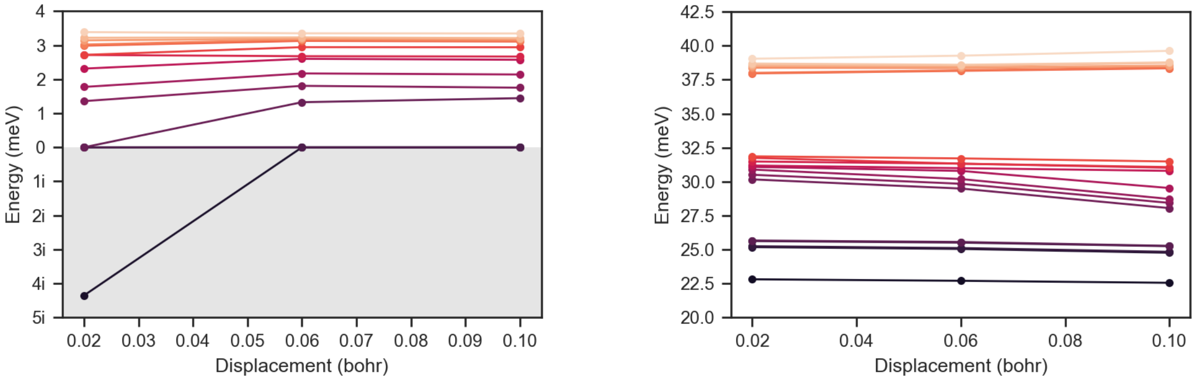

(a) Low energy phonons

(b) High energy phonons

Figure S7: Convergence with respect to displacement amplitude

Layered perovskites, however, contain both very heavy (Pb, I, Cs) and very light (H, C, N) atoms, which vibrate at different amplitudes. Thus, the suitable displacement for which we are in the harmonic regime may not be the same across all species. For low energy

modes, which predominantly involve vibrations of heavy atoms (Pb, I, Cs), we find converged frequencies (within 0.5 meV) up to 0.1 bohr. However, a displacement of 0.06 bohr is required to eliminate one imaginary frequency. On the other hand, high energy modes, which consist of internal vibrations of the organic ligands, start exhibiting large deviations in frequency beyond 0.06 bohr. In the end, we choose 0.06 bohr as a reasonable compromise between the inorganic and organic constituents. These results are shown in Figure S7.

We additionally compare the atom-resolved contributions to the Γ-point phonons obtained at 0.02 bohr, 0.06 bohr and 0.10 bohr, finding qualitative agreement across the range of tested displacement amplitudes (see Figure S8).

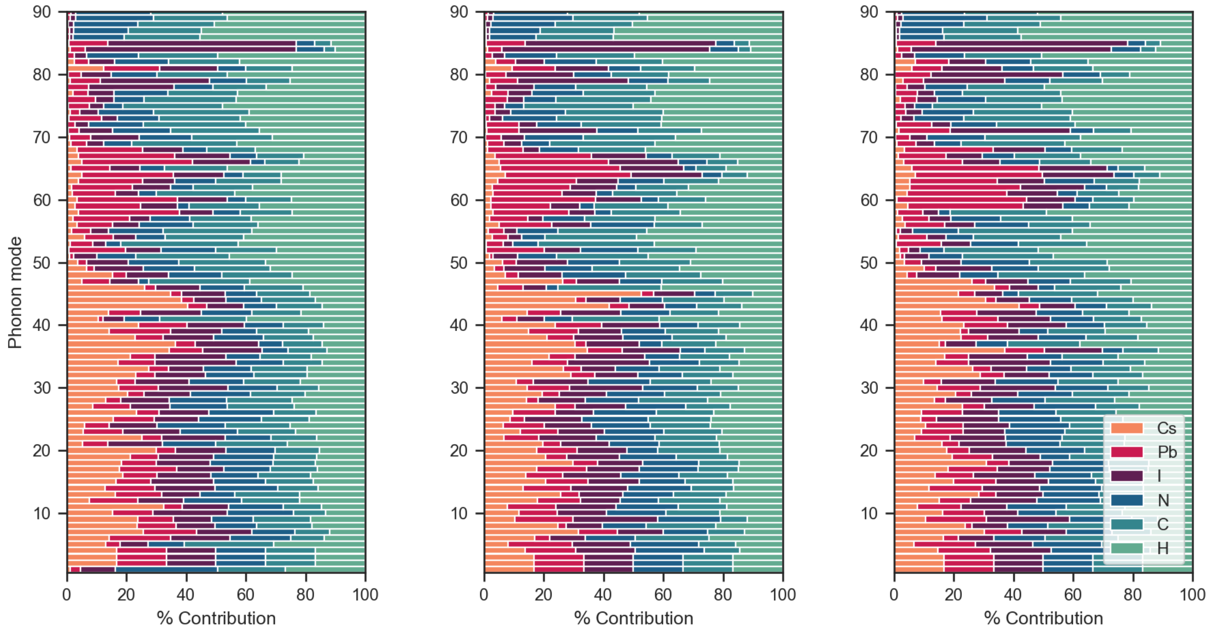

(a) d = 0.02 bohr (b) d = 0.06 bohr (c) d = 0.10 bohr

Figure S8: Projected contributions, by atom, to Γ-point phonon modes of $(HA)_2CsPb_2I_7$ obtained using various displacement amplitudes

As a final check, we compute the potential energy surface along characteristic phonon modes of the layered perovskite. These include mixed inorganic/organic modes, isolated vibrations of the lead-iodide octahedra, Cs-rattling, ligand bending and ligand internal molecular vibrations. We find that the potential energy curves were well-converged and well-described by quadratic functions across the range of displacement amplitudes tested

(see Figure S9). This further supports our use of the harmonic approximation to study layered perovskite systems. We note Figure S9a is not at the exact minima of the potential well, which we attribute to numerical noise arising from the chosen μ_β being large relative to the phonon mode's energy scale ($\sim 1\,\mathrm{meV}$) and eigendisplacement amplitude.

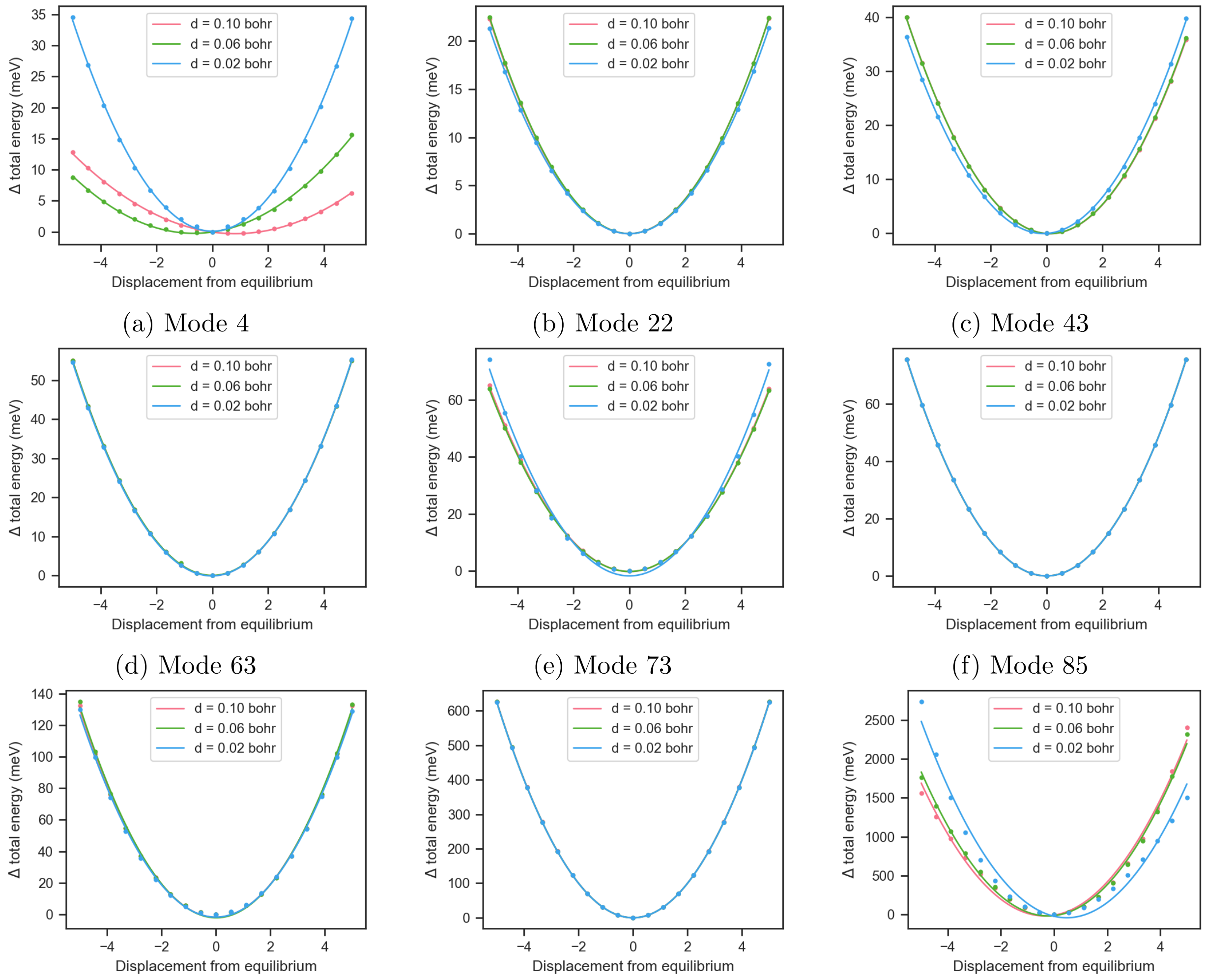

(a) Mode 4 (b) Mode 22 (c) Mode 43

(d) Mode 63 (e) Mode 73 (f) Mode 85

(g) Mode 100 (h) Mode 160 (i) Mode 315

Figure S9: Potential energy surfaces along characteristic phonon modes of $(HA)_2CsPb_2I_7$. Dots are computed total energies and lines are quadratic fits. Modes are 4) shearing of the perovskite subphase; 22) perovskite octahedral rotations; 43) Cs rattling in perovskite cage; 63) axial Pb-I stretching with off-center Pb displacement; 73) HA bending along N–C–C· backbone; 85) Pb-I octahedra breathing involving only five Pb-I bonds; 100) CH_3 and NH_3 symmetric rocking; 160) CH_3 and NH_3 asymmetric rocking; 315) NH_3 symmetric N–H stretching

Structural data for $(HA)_2(MA)Pb_2I_7$

Table S2: Lattice parameters for $(HA)_2(MA)Pb_2I_7$

**Compound name**		$\mathbf{(HA)_2MAPb_2I_7}$						
		DFT structures for various MA configurations				XRD measured lattice parameters		
**Functional**		Config. 1	Config. 2	Config. 3	Config. 4	Expt.[1]	Expt.[2]	Expt.[3]
**PBEsol**	a (Å)	8.695	8.714	8.694	8.675	8.695(3)	8.8062(8)	8.816(13)
	b (Å)	8.745	8.731	8.702	8.730	8.814(3)	8.9209(2)	8.929(2)
	c (Å)	47.006	47.122	47.614	48.045	45.146(16)	45.3552(2)	45.481(2)
	α (deg)	89.979	90.000	89.904	89.898	90	90	90
	β (deg)	97.191	96.945	95.940	94.813	100.030(5)	98.2088(8)	98.159(5)
	γ (deg)	90.294	90.000	89.818	89.832	90	90	90
	vol. (Å^3)	3546.41	3558.96	3583.15	3625.73	3407(2)	3526.56(13)	3544.0(17)
**PBE+TS**	a (Å)	8.739	8.791	8.756	8.766	8.695(3)	8.8062(8)	8.816(13)
	b (Å)	8.822	8.813	8.774	8.772	8.814(3)	8.9209(2)	8.929(2)
	c (Å)	45.530	45.300	45.498	45.424	45.146(16)	45.3552(2)	45.481(2)
	α (deg)	89.762	90.000	89.795	89.767	90	90	90
	β (deg)	101.42	101.69	100.73	101.36	100.030(5)	98.2088(8)	98.159(5)
	γ (deg)	90.179	90.000	90.079	90.279	90	90	90
	vol. (Å^3)	3440.88	3436.88	3434.23	3424.13	3407(2)	3526.56(13)	3544.0(17)
**PBEsol+TS**	a (Å)	8.616	8.630	8.618	8.629	8.695(3)	8.8062(8)	8.816(13)
	b (Å)	8.657	8.640	8.600	8.636	8.814(3)	8.9209(2)	8.929(2)
	c (Å)	45.167	45.058	45.22	44.863	45.146(16)	45.3552(2)	45.481(2)
	α (deg)	89.930	90.000	89.873	89.945	90	90	90
	β (deg)	102.22	102.17	101.28	101.60	100.030(5)	98.2088(8)	98.159(5)
	γ (deg)	90.245	90.000	90.049	89.838	90	90	90
	vol. (Å^3)	3292.4	3284.33	3286.60	3274.74	3407(2)	3526.56(13)	3544.0(17)

[1] Measured at 100 K by Ref. 2
[2] Measured at 298 K by Ref. 25
[3] Measured at 300 K by Ref. 26

Additional views of crystal structures

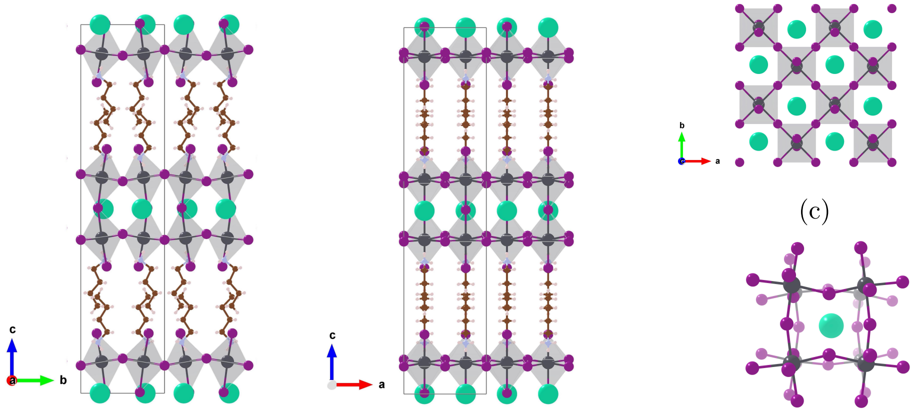

(a) (b) (c) (d)

Orthorhombic $(BA)_2(Cs)Pb_2I_7$

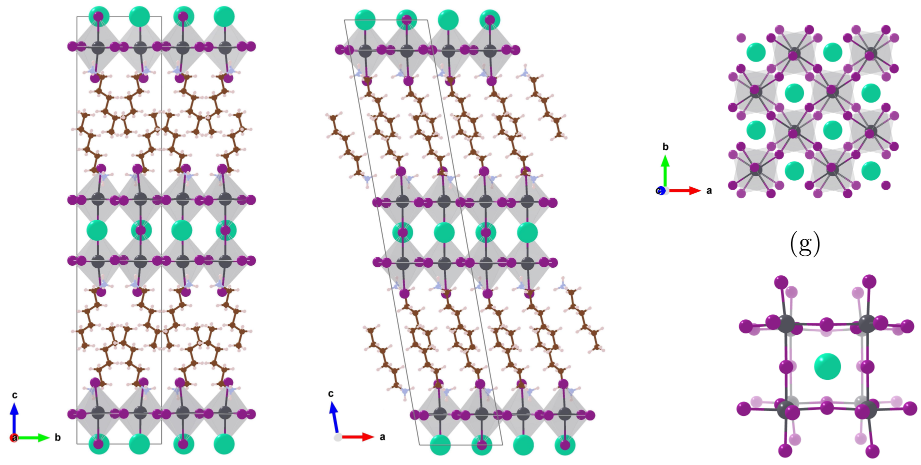

(e) (f) (g) (h)

Monoclinic $(HA)_2(Cs)Pb_2I_7$

Figure S10: Relaxed crystal structures for $(BA)_2(Cs)Pb_2I_7$ and $(HA)_2(Cs)Pb_2I_7$ obtained using PBEsol. (a, b, e, f) Sideviews showing alternating perovskite and organic subphases. (c, g) Top view of the perovskite layer showing Cs atoms embedded in lead-iodide framework. (d, h) Lead-iodide "cage" containing Cs at the A-site. Colors are purple (I), grey (Pb), brown (C), pink (N) and white (H), cyan (Cs).

Discrepancies between DFT and experimental structures

We note there are some structural features for which even PBE+TS, PBEsol+TS and PBE+MBD differ significantly from the experimental reference values. In both the BA- and HA-based systems, the Pb-I-Pb bond angles tend to be significantly smaller. Also, for the BA-based system, all benchmarked functionals predict a more rectangular base for the unit cell (b is 6–10% larger than a) compared to experimental data (b is only 1% larger than a). We attribute these discrepancies to temperature effects. In a study of n=1 $(BA)_2CsPb_2Br_7$, a similar layered perovskite, Ref. 27 report increasing differences in a and b lattice constants with decreasing temperature. In other words, the base of the unit cell is more rectangular at lower temperatures. Additionally, the lead-iodide cage becomes more distorted with decreasing temperature due to smaller Pb–I–Pb bond angles. Since our calculations were run at 0 K, but the experimental structures were measured at higher temperatures, we expect to see the observed discrepancies.

However, some Pb–I bond lengths in the calculated 0 K structure are *larger* than the higher temperature experimental measurements. This is the opposite of what is expected from thermal expansion. We attribute this to finite-size effects. Bulk halide perovskites are known to exhibit significant dynamical disorder at room temperature. Their true structure consists of a network of low-symmetry local motifs that forms an average structure of high symmetry.[28,29] As we are working only with the primitive cell, we may be artificially constraining the amount of disorder across adjacent lead-iodide octahedra, resulting in some Pb–I bond lengths being ±2% away from the experimental values.

Another discrepancy is that all GGA with dispersion correction methods overestimate the ligand bilayer thickness in $(BA)_2(Cs)Pb_2I_7$ by around 1 Å. This is driven primarily by less overlap (or "interlocking") between the aliphatic CH_3 tails compared to experiment. Since dispersion dominates the interaction between the ligands' CH_3 tails, the inaccuracy is likely linked to an imperfect dispersion correction scheme. We note that this discrepancy exists for both the pair-wise and many-body schemes, so we cannot attribute it to the lack of many-

body interactions. Interestingly, we did not find the same discrepancy for $(HA)_2(Cs)Pb_2I_7$. We attribute this to differences in the phase of the ligand bilayer. Work by Ref. 26 and Ref. 30 on analogous layered perovskites showed that the transition from the low temperature (monoclinic or triclinic) to the high temperature phase (orthorhombic) is associated with a partial melting of the organic bilayer. In other words, the organic bilayer in orthorhombic $(BA)_2(MA)Pb_2I_7$ is partially melted and therefore disordered, while the organic bilayer in monoclinic $(HA)_2(MA)Pb_2I_7$ is not. It is possible that the dispersion correction schemes benchmarked are less accurate for weakly-bonded molecular liquids.

An extended discussion of Γ-point phonon modes

In the main text, we focus our discussion on vibrational modes involving coupled motions of the perovskite and ligand subphases and vibrational modes unique to quasi-2D perovskites. Here, we focus our discussion on the normal modes analogous to those of the reference systems: 3D perovskites, the HA molecule and HA molecular crystal.

Stretching of Pb−I bonds

These are analogous to Pb−I bond stretching modes in cubic 3D perovskites. However, due to the anisotropy in the quasi-2D perovskite, we distinguish between *equatorial* (in the a,b-plane) and *axial* (along the c-axis) Pb-I bonds. Each lead-iodide octahedron contains four equatorial and two axial bonds.

For *equatorial* Pb−I bonds, adjacent lead-iodide octahedra in the same perovskite layer vibrate out-of-phase with each other. There are two versions here: in the first version, illustrated in Figure S11a, two Pb−I bonds are elongating while the other two are contracting. This is analogous to the 12.2 meV (98.1 cm^{-1}) in tetragonal $MAPbI_3$ identified in.[31] In the second version, illustrated in Figure S11b, all four Pb−I bonds are elongating at the same time. This is analogous to the B_{2g} mode at 13.5 meV (104.9 cm^{-1}) identified in.[32]

For *axial* Pb−I bonds, adjacent lead-iodide octahedra in the same perovskite layer may vibrate in-phase *or* out-of-phase with each other. The mode in Figure S11c involves out-of-phase axial Pb−I stretching. It resembles the B_{3g} mode at 11.1 meV (90.0 cm^{-1}) identified in,[32] and at 11.2 meV (90.6 cm^{-1}) in.[31] We note that Ref. 33 found that axial Pb−I bond stretching coupled to the excited electronic state, forming a polaron. Meanwhile, the mode in Figure S11d shows in-phase stretching for all octahedra in the same layer. This resembles the B_{1g} mode at 12.2 meV (98.8 cm^{-1}) identified in.[32] We note that the energies for analogous vibrations in our quasi-2D system are similar, although not identical, to their 3D counterparts. The relatively small difference in energy is likely due to coupling with vibrations of Cs or ligands in our quasi-2D system.

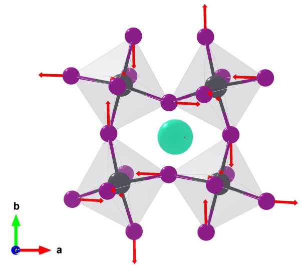

(a) Mode 79 (13.4 meV)

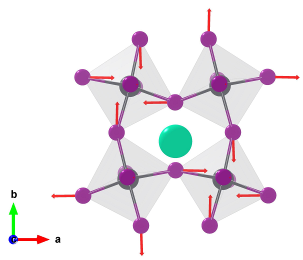

(b) Mode 84 (14.7 meV)

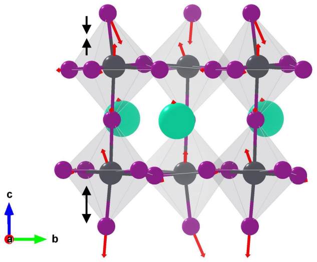

(c) Mode 56 (10.1 meV)

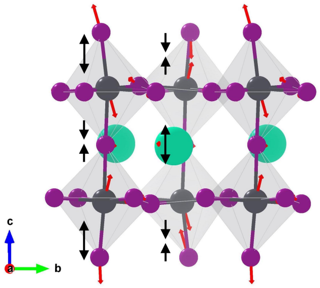

(d) Mode 63 (10.8 meV)

Figure S11: Vibrations involving stretching of equatorial and axial Pb-I bonds

Bending, rocking and twisting of I−Pb−I bonds

Bending, rocking and twisting of I−Pb−I bonds result in various symmetric and asymmetric distortions to equilibrium I−Pb−I angles and therefore the PbI_6 octahedron shape. In Figure S12a, *cis*-I−Pb−I bonds show symmetric scissoring. This results in an overall contraction of the lead-iodide cage around central Cs cation (and an expansion of the lead-iodide cage for the neighboring Cs cation). In Figure S12b, the pattern of I−Pb−I bending in the top equatorial plane (dark shading) is out-of-phase with that of the bottom equatorial plane (faded shading). Finally, in Figure S12c, I−Pb−I bonds bend in the *c*-axis and couple to displacements of Pb atoms from their equilibrium positions at the center of the lead-iodide octahedra. These motions are further coupled to the Cs cation at the A-site displacing along the *c*-axis. Consistent with results for 3D $MAPbI_3$,[31] I−Pb−I bending and rocking modes up to ∼10 meV generally involve displacements of Pb atoms, while those above ∼10 meV generally involve only displacements of I atoms.

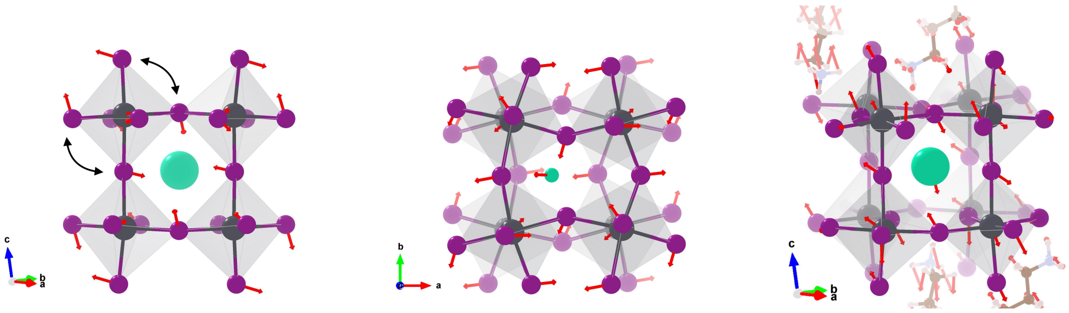

(a) Mode 27 (2.7 meV) (b) Mode 29 (2.9 meV) (c) Mode 10 (5.3 meV)

Figure S12: Phonon modes involving a mix of bending and rocking of I−Pb−I bonds

Rattling of the A-site cation inside the perovskite cage

The displacements of Cs cations are coupled with deformations of the surrounding lead-halide cage, specifically to the axis of motion of equatorial I atoms. The vibration of the perovskite subphase also tugs the ligands along as rigid-bodies. In Figure S13a, adjacent Cs cations move out-of-phase, while in Figure S13b, Cs cations move in-phase.

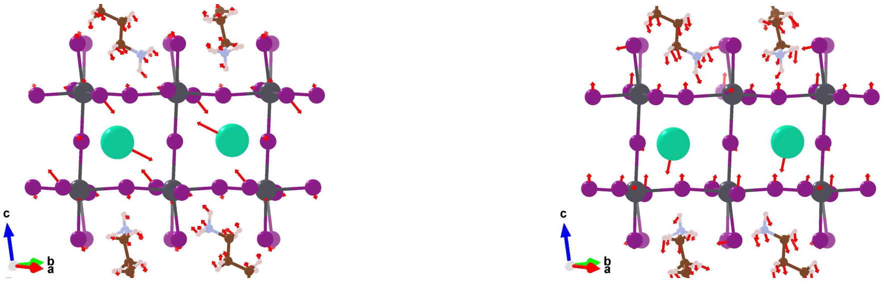

(a) Mode 43 (7.2 meV) (b) Mode 47 (8.2 meV)

Figure S13: Rattling of Cs within the lead-iodide cage

Rotation and tilt of lead-iodide octahedra

In this work, we distinguish between *rotating*, where the octahedra rotates about the c-axis, and *tilting*, where the octahedra tilts in the a,c or b,c planes. We note that in cubic 3D perovskites, these two descriptions are interchangeable.

For *rotating*, the two octahedral layers stacked along the c-axis may rotate in-phase or out-of-phase. The in-phase variation is illustrated in Figure S14a while the out-of-phase variation is illustrated in Figure S14b.

For tilting, there are also two variations. In the first (Figure S14b), all octahedra in the same equatorial plane (a,b-plane) tilt in-phase. This leads to opposing "shear"-like motions of the two octahedral layers, accompanied by displacements of the A-site cations. Meanwhile, in the second version (Figure S14c), lead-iodide octahedra in the same octahedral plane are out-of-phase with each other.

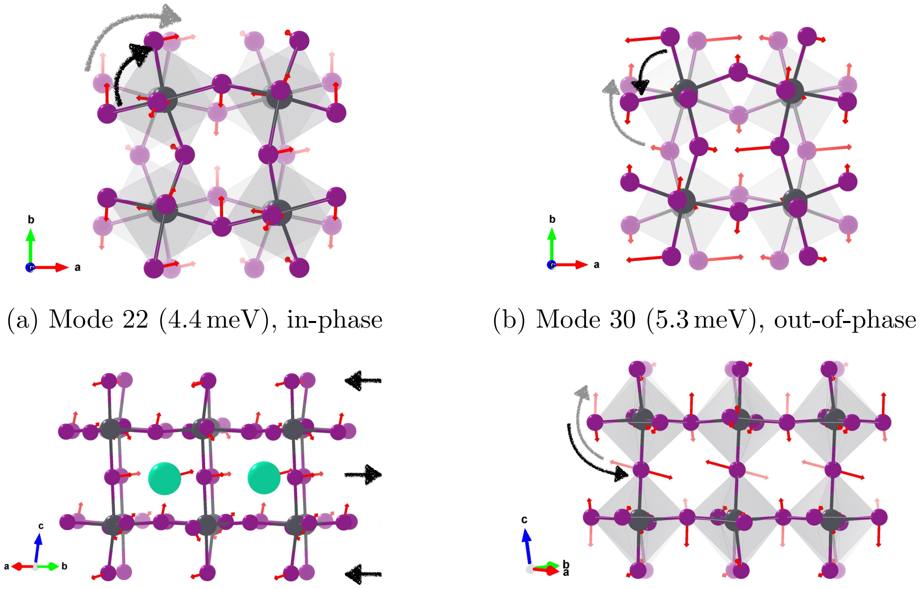

(a) Mode 22 (4.4 meV), in-phase

(b) Mode 30 (5.3 meV), out-of-phase

(c) Mode 8 (2.7 meV), in-phase

(d) Mode 9 (2.9 meV), out-of-phase

Figure S14: Rotation and tilt modes of lead-iodide octahedra.

Ligand-dominated modes

There are many low energy phonon modes where the dominant contribution comes from the hexylammonium (HA) ligands. We discuss these modes in the context of computed normal modes for two reference systems: (i) a free HA molecule and (ii) the HA molecular crystal. In general, in the quasi-2D perovskite, the HA molecule and the molecular crystal, most low energy vibrational modes involve rotation and libration of successive C–N and C–C bonds along the ligand backbone. There is additionally a smaller contribution from scissoring, stretching and rocking of C–H and N–H bonds. However, the vibration amplitude is more uniform along the ligand for the free molecule and molecular crystal. This is because, in

the quasi-2D perovskite, the ligands' ammonium heads are fixed through a mix of ionic and hydrogen bonding. Thus the ligands in quasi-2D perovskites show larger vibration amplitudes at the (less-fixed) hydrocarbon tails.

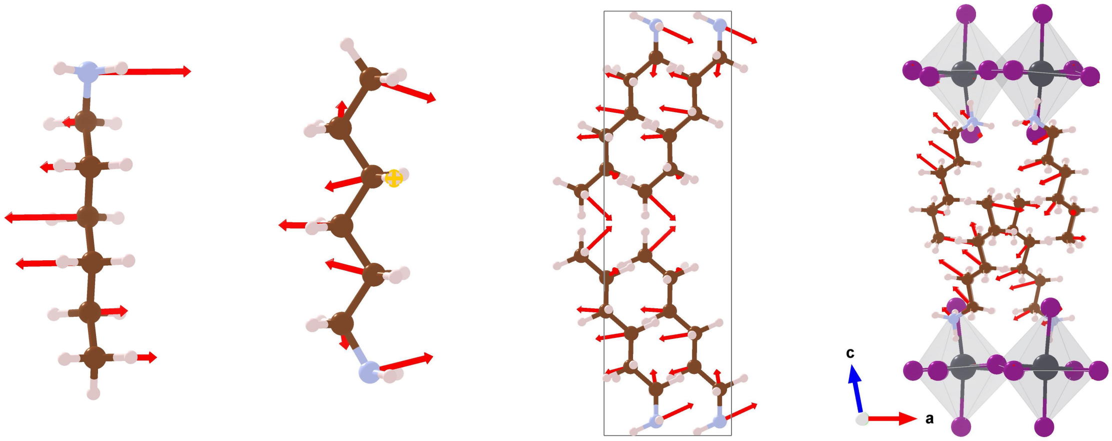

(a) 7.0 meV (b) 11.2 meV (c) 11.3 meV (d) Mode 73 (12.2 meV)

Figure S15: Vibrational modes involving "bending" of the ligand backbone in (a), (b) the isolated HA molecule; (c) the HA molecular crystal; (d) the layered perovskite $(HA)_2CsPb_2I_7$. Eigenvectors on hydrogen atoms have been removed for clarity

We illustrate some of these similarities and differences in Figure S15. In the free HA molecule, we observe two versions of successive librations of C−C bonds along the ligand backbone, which "curl up" the molecule. The first is Figure S15a, where the bending plane of motion is *perpendicular* to the mirror plane of the molecule. The second is Figure S15b, where the bending plane of motion lies within the mirror plane of the molecule. In the HA molecular crystal, there are clusters of analogous modes at similar frequencies to the free molecule modes. For example, the molecular crystal mode in Figure S15c is analogous to that of Figure S15b. Finally, in the quasi-2D perovskite, there is a similar phonon mode where the ligand backbone bends along the mirror plane. However, the amplitude of vibration is smaller at the ammonium head compared to the hydrocarbon tail. We attribute this to the ammonium head being more constrained in space due to the surrounding perovskite, and the ammonium head being positively charged, and therefore being more strongly bound via ionic interactions with the perovskite subphase.

We also compare our results for the NH_3^+ bending modes to that of Lavan et al.[34]. In their work, they obtain a symmetric NH_3^+ bending mode at 1449.8 cm^{-1} and asymmetric NH_3^+ bending modes at 1500.6 cm^{-1} and 1536.4 cm^{-1} identified for the $n = 1$ perovskite $(HA)_2PbI_4$. In our work, for $(HA)_2CsPb_2I_7$, we identify symmetric modes at 180.2 meV (1454.1 cm^{-1}) and various asymmetric modes ranging from 180.9 meV to 193.4 meV (1459.6 cm^{-1} to 1559.9 cm^{-1}). For $(HA)_2(MA)Pb_2I_7$, we identify symmetric modes for HA at 179.3 meV and 179.6 meV (1446.4 cm^{-1} and 1448.8 cm^{-1}) and for MA at 180.6 meV and 180.7 meV (1456.6 cm^{-1} and 1457.3 cm^{-1}). In $(HA)_2(MA)Pb_2I_7$, the asymmetric modes for HA range from 179.3 meV to 192.9 meV (1446.5 cm^{-1} to 1556.0 cm^{-1}), while the two asymmetric modes for MA are at 189.3 (1526.5 cm^{-1}). In agreement with Lavan et al.[34], we also find that the NH_3^+ bending modes are shifted to lower energies compared to in an isolated HA^+ cation fragment. However, we did not find a clear separation in frequencies between the HA-only and MA-only modes. As we did not compute phonon modes for perovskites containing other halogen or metal atoms, we cannot comment on the usefulness of Raman spectra in this frequency range for probing different quasi-2D perovskites with different ions in the inorganic lattice.

IR spectra

We provide the predicted IR spectra over all frequencies for $(HA)_2CsPb_2I_7$ for reference (the low energy spectra are shown in the main text). We note that the higher energy peaks correspond well to typical IR-active vibrational modes of organic molecules.

Atom-resolved contributions of all modes

We show the atom-resolved contributions for all 336 Γ-point phonon modes of $(HA)_2CsPb_2I_7$ for reference (the first 100 modes are shown in the main text).

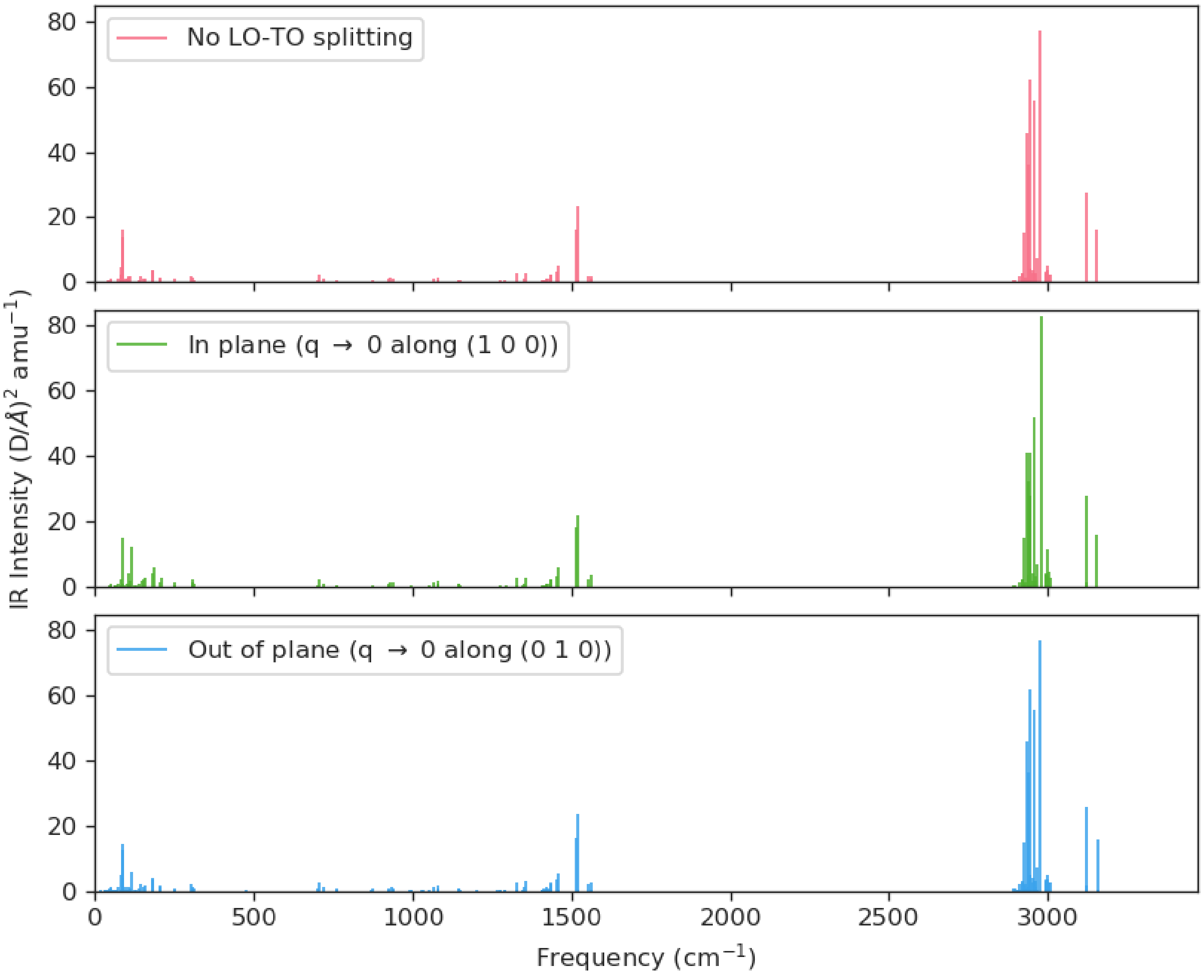

Figure S16: Predicted IR spectra for $(HA)_2CsPb_2I_7$.

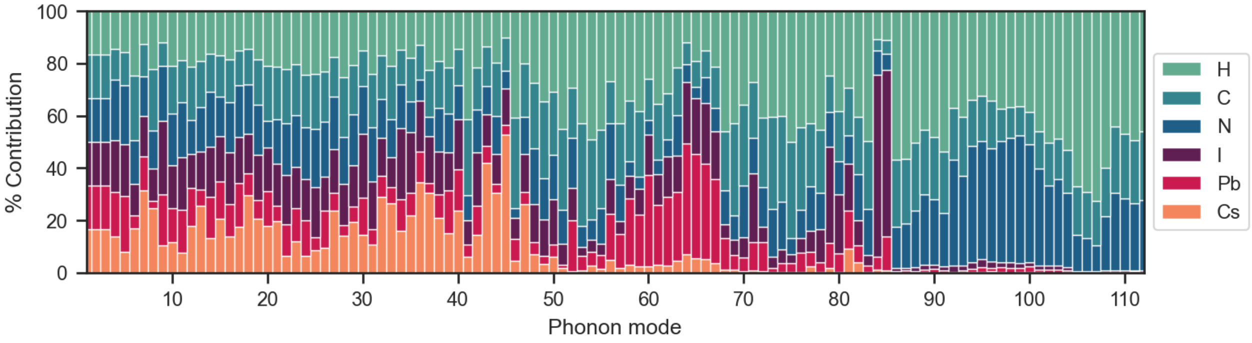

(a) Modes 1-112

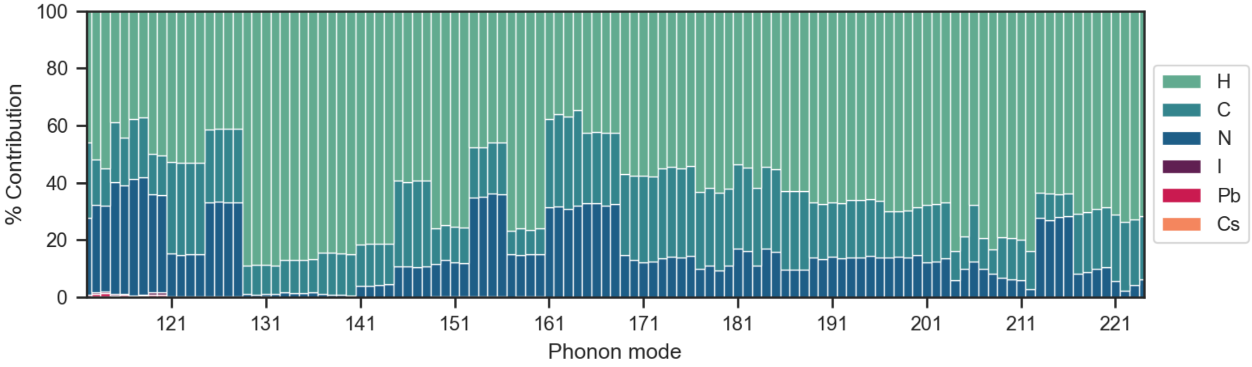

(b) Modes 113-224

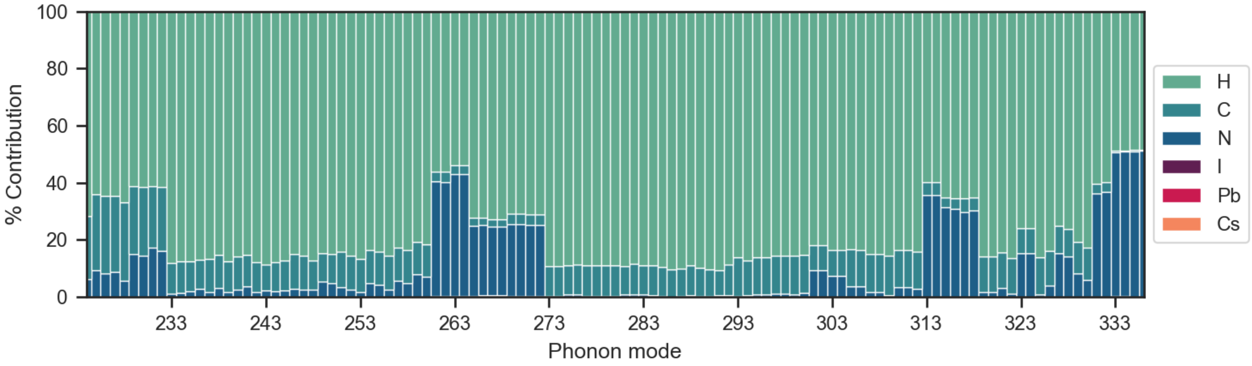

(c) Modes 225-336

Figure S17: Atom-resolved contributions for all Γ-point phonon modes of $(HA)_2CsPb_2I_7$. Note: relative contributions are scaled by the number of atoms in the system

More phonon dispersion

We show the full phonon dispersion spectrum, as well as projection to the ligand (HA) subphase and just the A-site cation (Cs) for the low energy phonon spectrum.

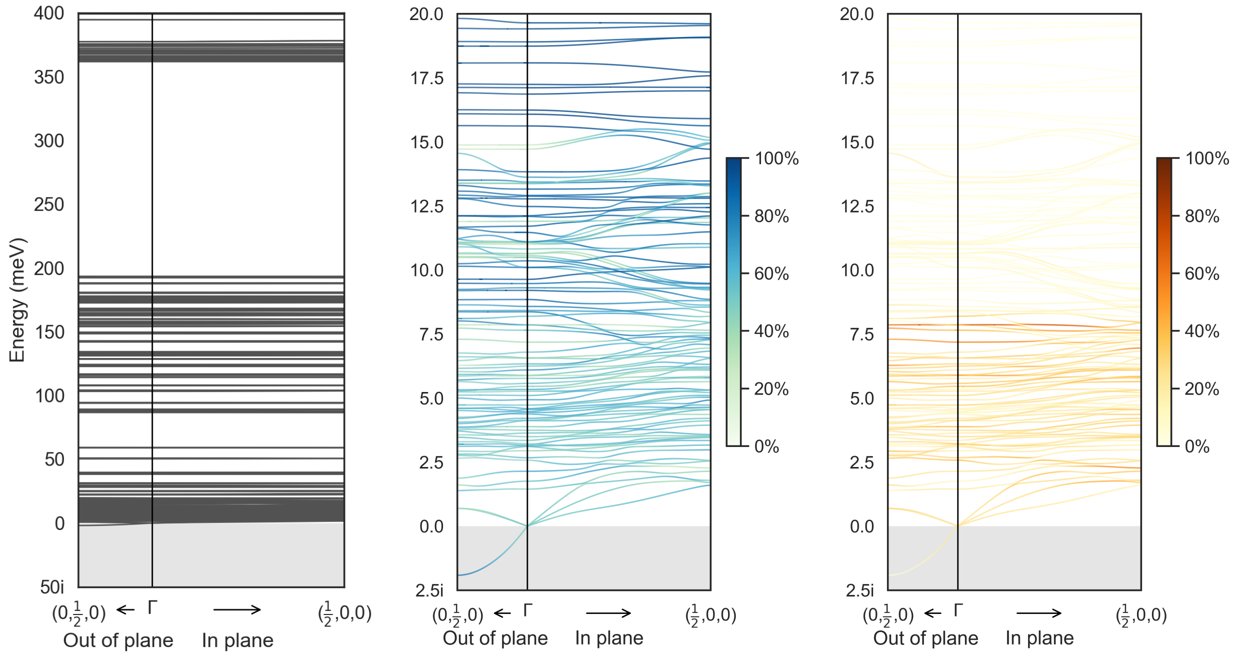

(a) (b) (c)

Figure S18: Phonon dispersion for $(HA)_2CsPb_2I_7$ (a) for the full energy scale (b) projected to the ligand subphase and (c) projected to Cs at A-sites

Zone boundary soft mode

Additionally, we sample total energies along the slight soft mode at the out-of-plane zone boundary and confirm that the potential energy well is highly harmonic.

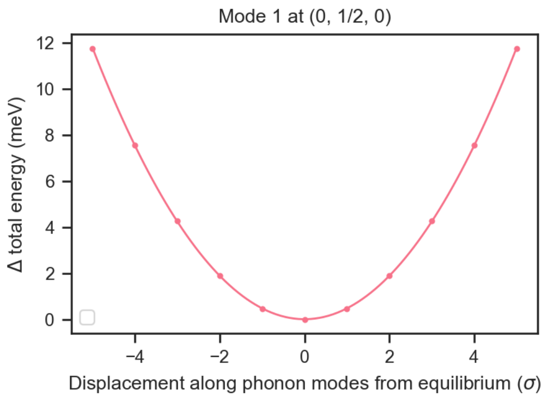

Figure S19: Potential energy well associated with the out-of-plane zone boundary soft mode

References

(1) Stoumpos, C. C.; Cao, D. H.; Clark, D. J.; Young, J.; Rondinelli, J. M.; Jang, J. I.; Hupp, J. T.; Kanatzidis, M. G. Ruddlesden–Popper Hybrid Lead Iodide Perovskite 2D Homologous Semiconductors. *Chemistry of Materials* **2016**, *28*, 2852–2867.

(2) Fu, Y.; Hautzinger, M. P.; Luo, Z.; Wang, F.; Pan, D.; Aristov, M. M.; Guzei, I. A.; Pan, A.; Zhu, X.; Jin, S. Incorporating Large A Cations into Lead Iodide Perovskite Cages: Relaxed Goldschmidt Tolerance Factor and Impact on Exciton–Phonon Interaction. *ACS Central Science* **2019**, *5*, 1377–1386.

(3) Bjorkman, T. CIF2Cell: Generating geometries for electronic structure programs. *Computer Physics Communications* **2011**, *182*, 1183 – 1186.

(4) Dhanabalan, B.; Leng, Y.-C.; Biffi, G.; Lin, M.-L.; Tan, P.-H.; Infante, I.; Manna, L.; Arciniegas, M. P.; Krahne, R. Directional Anisotropy of the Vibrational Modes in 2D-Layered Perovskites. *ACS Nano* **2020**, *14*, 4689–4697.

(5) Shannon, R. D. Revised effective ionic radii and systematic studies of interatomic distances in halides and chalcogenides. *Acta Crystallographica Section A* **1976**, *32*, 751–767.

(6) Park, N.-G. Perovskite solar cells: an emerging photovoltaic technology. *Materials Today* **2015**, *18*, 65–72.

(7) Hohenberg, P.; Kohn, W. Inhomogeneous Electron Gas. *Phys. Rev.* **1964**, *136*, B864–B871.

(8) Kohn, W.; Sham, L. J. Self-Consistent Equations Including Exchange and Correlation Effects. *Phys. Rev.* **1965**, *140*, A1133–A1138.

(9) Clark, S. J.; Segall, M. D.; Pickard, C. J.; Hasnip, P. J.; Probert, M. I. J.; Refson, K.;

Payne, M. C. First principles methods using CASTEP. *Zeitschrift für Kristallographie - Crystalline Materials* **2005**, *220*, 567–570.

(10) Vanderbilt, D. Soft self-consistent pseudopotentials in a generalized eigenvalue formalism. *Phys. Rev. B* **1990**, *41*, 7892–7895.

(11) Ceperley, D. M.; Alder, B. J. Ground State of the Electron Gas by a Stochastic Method. *Phys. Rev. Lett.* **1980**, *45*, 566–569.

(12) Perdew, J. P.; Zunger, A. Self-interaction correction to density-functional approximations for many-electron systems. *Phys. Rev. B* **1981**, *23*, 5048–5079.

(13) Perdew, J. P.; Burke, K.; Ernzerhof, M. Generalized Gradient Approximation Made Simple. *Phys. Rev. Lett.* **1996**, *77*, 3865–3868.

(14) Perdew, J. P.; Ruzsinszky, A.; Csonka, G. I.; Vydrov, O. A.; Scuseria, G. E.; Constantin, L. A.; Zhou, X.; Burke, K. Restoring the Density-Gradient Expansion for Exchange in Solids and Surfaces. *Phys. Rev. Lett.* **2008**, *100*, 136406.

(15) Ortmann, F.; Bechstedt, F.; Schmidt, W. G. Semiempirical van der Waals correction to the density functional description of solids and molecular structures. *Physical Review B* **2006**, *73*, 205101.

(16) Grimme, S. Semiempirical GGA-type density functional constructed with a long-range dispersion correction. *Journal of Computational Chemistry* **2006**, *27*, 1787–1799.

(17) Tkatchenko, A.; Scheffler, M. Accurate Molecular Van Der Waals Interactions from Ground-State Electron Density and Free-Atom Reference Data. *Physical Review Letters* **2009**, *102*, 073005.

(18) Tkatchenko, A.; DiStasio, R. A.; Car, R.; Scheffler, M. Accurate and Efficient Method for Many-Body van der Waals Interactions. *Phys. Rev. Lett.* **2012**, *108*, 236402.

(19) Ambrosetti, A.; Reilly, A. M.; DiStasio, J., Robert A.; Tkatchenko, A. Long-range correlation energy calculated from coupled atomic response functions. *The Journal of Chemical Physics* **2014**, *140*, 18A508.

(20) Marronnier, A.; Lee, H.; Geffroy, B.; Even, J.; Bonnassieux, Y.; Roma, G. Structural Instabilities Related to Highly Anharmonic Phonons in Halide Perovskites. *The Journal of Physical Chemistry Letters* **2017**, *8*, 2659–2665.

(21) Klarbring, J.; Hellman, O.; Abrikosov, I. A.; Simak, S. I. Anharmonicity and Ultralow Thermal Conductivity in Lead-Free Halide Double Perovskites. *Physical Review Letters* **2020**, *125*, 045701.

(22) Kunc, K.; Martin, R. M. Ab Initio Force Constants of GaAs: A New Approach to Calculation of Phonons and Dielectric Properties. *Phys. Rev. Lett.* **1982**, *48*, 406–409.

(23) Refson, K.; Tulip, P. R.; Clark, S. J. Variational density-functional perturbation theory for dielectrics and lattice dynamics. *Phys. Rev. B* **2006**, *73*, 155114.

(24) Momma, K.; Izumi, F. VESTA for three-dimensional visualization of crystal, volumetric and morphology data. *Journal of Applied Crystallography* **2011**, *44*, 1272–1276.

(25) Spanopoulos, I.; Hadar, I.; Ke, W.; Tu, Q.; Chen, M.; Tsai, H.; He, Y.; Shekhawat, G.; Dravid, V. P.; Wasielewski, M. R.; et al., Uniaxial Expansion of the 2D Ruddlesden–Popper Perovskite Family for Improved Environmental Stability. *Journal of the American Chemical Society* **2019**, *141*, 5518–5534.

(26) Paritmongkol, W.; Dahod, N. S.; Stollmann, A.; Mao, N.; Settens, C.; Zheng, S.-L.; Tisdale, W. A. Synthetic Variation and Structural Trends in Layered Two-Dimensional Alkylammonium Lead Halide Perovskites. *Chemistry of Materials* **2019**, *31*, 5592–5607.

(27) Chen, H.; Lin, J.; Kang, J.; Kong, Q.; Lu, D.; Kang, J.; Lai, M.; Quan, L. N.; Lin, Z.;

Jin, J.; et al., Structural and spectral dynamics of single-crystalline Ruddlesden-Popper phase halide perovskite blue light-emitting diodes. *Science Advances* **2020**, *6*, eaay4045.

(28) van de Goor, T. W. J.; Liu, Y.; Feldmann, S.; Bourelle, S. A.; Neumann, T.; Winkler, T.; Kelly, N. D.; Liu, C.; Jones, M. A.; Emge, S. P.; et al., Impact of Orientational Glass Formation and Local Strain on Photo-Induced Halide Segregation in Hybrid Metal-Halide Perovskites. *The Journal of Physical Chemistry C* **2021**, *125*, 15025–15034.

(29) Zhao, X.-G.; Dalpian, G. M.; Wang, Z.; Zunger, A. Polymorphous nature of cubic halide perovskites. *Phys. Rev. B* **2020**, *101*, 155137.

(30) Dahod, N. S.; Paritmongkol, W.; Stollmann, A.; Settens, C.; Zheng, S.-L.; Tisdale, W. A. Melting Transitions of the Organic Subphase in Layered Two-Dimensional Halide Perovskites. *The Journal of Physical Chemistry Letters* **2019**, *10*, 2924–2930.

(31) Brivio, F.; Frost, J. M.; Skelton, J. M.; Jackson, A. J.; Weber, O. J.; Weller, M. T.; Goñi, A. R.; Leguy, A. M. A.; Barnes, P. R. F.; Walsh, A. Lattice dynamics and vibrational spectra of the orthorhombic, tetragonal, and cubic phases of methylammonium lead iodide. *Phys. Rev. B* **2015**, *92*, 144308.

(32) Pérez-Osorio, M. A.; Milot, R. L.; Filip, M. R.; Patel, J. B.; Herz, L. M.; Johnston, M. B.; Giustino, F. Vibrational Properties of the Organic–Inorganic Halide Perovskite $(CH_3NH_3)PbI_3$ from Theory and Experiment: Factor Group Analysis, First-Principles Calculations, and Low-Temperature Infrared Spectra. *The Journal of Physical Chemistry C* **2015**, *119*, 25703–25718.

(33) Park, M.; Neukirch, A. J.; Reyes-Lillo, S. E.; Lai, M.; Ellis, S. R.; Dietze, D.; Neaton, J. B.; Yang, P.; Tretiak, S.; Mathies, R. A. Excited-state vibrational dynamics toward the polaron in methylammonium lead iodide perovskite. *Nature Communications* **2018**, *9*, 2525.

(34) Lavan, S. N.; Sanni, A. M.; Rury, A. S.; Liu, Z.-F. Characterization of the Ammonium Bending Vibrations in Two-Dimensional Hybrid Lead-Halide Perovskites from Raman Spectroscopy and First-Principles Calculations. *The Journal of Physical Chemistry C* **2021**, *125*, 223–236.
